# Biopolymeric Insulin Membranes for Antimicrobial, Antioxidant, and Wound Healing Applications

**DOI:** 10.3390/pharmaceutics16081012

**Published:** 2024-07-30

**Authors:** Rocío Aguilar-Vázquez, Alejandra Romero-Montero, María L. Del Prado-Audelo, Lizbeth Cariño-Calvo, Manuel González-Del Carmen, Pablo Adrián Vizcaíno-Dorado, Isaac Hiram Caballero-Florán, Sheila Iraís Peña-Corona, Juan Isaac Chávez-Corona, María Josefa Bernad-Bernad, Jonathan J. Magaña, Hernán Cortés, Gerardo Leyva-Gómez

**Affiliations:** 1 Departamento de Farmacia, Facultad de Química, Universidad Nacional Autónoma de México, Ciudad de México 04510, Mexico; rocioaguilar90@hotmail.com (R.A.-V.); alejandra.romero.montero@outlook.com (A.R.-M.); sheila.ipc@live.com (S.I.P.-C.); juan.isaac.chavez@gmail.com (J.I.C.-C.); bernadf@comunidad.unam.mx (M.J.B.-B.); 2 Tecnologico de Monterrey, Escuela de Ingenieria y Ciencias, Campus Ciudad de Mexico, Ciudad de Mexico 14380, Mexico; luisa.delprado@tec.mx (M.L.D.P.-A.); icaballero@cinvestav.mx (I.H.C.-F.); magana.jj@tec.mx (J.J.M.); 3 Facultad de Ciencias Químicas, Universidad Veracruzana, Orizaba 94340, Mexico; lcarino@uv.mx; 4 Facultad de Medicina, Universidad Veracruzana, Ciudad Mendoza 94740, Mexico; manugonzalez@uv.mx; 5 Laboratorio de Medicina Genómica, Departamento de Genómica, Instituto Nacional de Rehabilitación Luis Guillermo Ibarra Ibarra, Ciudad de México 14389, Mexico; adrian_vizcaino@hotmail.com (P.A.V.-D.); hcortes_c@hotmail.com (H.C.); 6 Laboratorio de Investigación y Posgrado en Tecnología Farmacéutica, Universidad Nacional Autónoma de México-FESC, Campus 1, Cuautitlán Izcalli 54714, Mexico; 7 Departamento de Fisiología, Biofísica y Neurociencias, Centro de Investigación y de Estudios Avanzados del Instituto Politécnico Nacional, Ciudad de México 04510, Mexico

**Keywords:** drug repositioning, insulin, polymeric membranes, poloxamer 188, antioxidant, antibacterial activity, in vitro, in vivo, wound healing

## Abstract

Delayed wound healing increases the wound’s vulnerability to possible infections, which may have lethal outcomes. The treatments available can be effective, but the urgency is not fully encompassed. The drug repositioning strategy proposes effective alternatives for enhancing medical therapies for chronic diseases. Likewise, applying wound dressings as biodegradable membranes is extremely attractive due to their ease of application, therapeutic effectiveness, and feasibility in industrial manufacturing. This article aims to demonstrate the pleiotropic effects during insulin repositioning in wound closure by employing a biopolymeric membrane-type formulation with insulin. We prepared biopolymeric membranes with sodium alginate cross-linked with calcium chloride, supported in a mixture of xanthan gum and guar gum, and plasticized with glycerol and sorbitol. Human insulin was combined with poloxamer 188 as a protein stabilizing agent. Our investigation encompassed physicochemical and mechanical characterization, antioxidant and biological activity through antibacterial tests, cell viability assessments, and scratch assays as an in vitro and in vivo wound model. We demonstrated that our biopolymeric insulin membranes exhibited adequate manipulation and suitable mechanical resistance, transparency, high swelling capability (1100%), and 30% antioxidant activity. Furthermore, they exhibited antibacterial activity (growth inhibition of *S. aureus* at 85% and *P. aeruginosa* at 75%, respectively), and insulin promoted wound closure in vitro with a 5.5-fold increase and 72% closure at 24 h. Also, insulin promoted in vivo wound closure with a 3.2-fold increase and 92% closure at 10 days compared with the groups without insulin, and this is the first report that demonstrates this therapeutic effect with two administrations of 0.7 IU. In conclusion, we developed a multifunctional insulin-loaded biopolymeric membrane in this study, with the main activity derived from insulin’s role in wound closure and antioxidant activity, augmented by the antimicrobial effect attributed to the polymer poloxamer 188. The synergistic combination of excipients enhances its usefulness and highlights our innovation as a promising material in wound healing materials.

## 1. Introduction

Chronic wounds (CWs) represent a silent epidemic in the world associated with co-morbidities such as diabetes and obesity [1]. Around 40 million people are afflicted with CWs, significantly diminishing the quality of life (QoL) of nearly 2.5% of the population. Also, the World Health Organization data suggest that antimicrobial resistance accounts for 700,000 mortalities annually [2], predominantly due to pathogenic bacteria such as *Staphylococcus aureus*, *Pseudomonas aeruginosa*, *Enterococcus faecalis*, and *Proteus mirabilis* [3]. Therefore, CW care has a significant economic impact of about USD 28.1–96.8 billion [4], according to the Global Wound Care Market in 2016, and an increase to USD 26 billion in 2023 [5]. Addressing this morbidity and optimizing wound care necessitate the implementation of comprehensive prevention and treatment protocols [6] to improve the QoL of patients through efficient treatments and decrease hospital stays [7].

The CW management strategies range from traditional wound care treatment (dressing-free therapy) to innovative approaches (skin grafting and dressing therapies) for wound healing, depending on the type of injury [5]. One of the traditional wound care methods is biological therapy, which uses growth factors [5,8] or protein treatments to accelerate healing. Insulin, a peptide hormone and growth factor comprising two chains of 24 amino acids, forms hexamers with zinc ion ligands for stabilizing [9]. Although insulin’s primary physiological activity is to modulate the blood glucose levels, some interesting studies have demonstrated its role in wound healing, restoring the integrity of skin damage via topical administration [10]. Topical insulin treatment improves wound healing by regulating oxidative stress, anti-inflammatory responses [10], keratinocyte migration, collagen deposition [11], and angiogenesis [12]. However, the current administration methods, such as subcutaneous injections, are painful and invasive [13]. Therefore, novel delivery systems extending the dosing intervals and ensuring protein stability are imperative, with innovations including microparticles [14], nanoparticles [15], microneedles [16], sponges [17], films [18], membranes [19], thermosensitive, polymeric and liposomal gels [20,21,22,23,24], and protein stabilizers as surfactants [25]. Insulin administration requires adjunctive excipients to maintain its stability and regulate its release. In this respect, polymer-based membranes loaded with bioactive agents are wound dressings suitable for CWs that could incorporate insulin [26]. Biodegradable polymeric and gum-derived membranes offer advantages such as biosafety, biocompatibility, swelling capability, semi-occlusive properties, transparency for monitoring wound closure, and flexibility [27]. From an economic perspective, biopolymeric membranes are cost-effective, align with circular economy principles, reutilize the natural metabolites from plastic-free materials, and are fully biodegradable and resorbable on the wound [28].

Biopolymers such as sodium alginate (SA), xanthan gum (XG), and guar gum (GG) are water-soluble polymers derived from natural sources [29] and undergo hydrolytic degradation in biological fluids [27]. SA is obtained from macroalgae; it is conformed by D-glucuronic (G) and D-mannuronic (M) moieties, having a high capability of adsorbing wound exudate and easily binding with a variety of metal ions (Ca^2+^) to cross-link bulk [30]. XG is an anionic polysaccharide biosynthesized by *Xanthomonas* bacteria; their chains are conformed by 1,4 D-glucose and 2:1 D-Mannose and D-Glucuronic acid, yielding viscous solutions that form elastic and adhesive materials [27]. Also, GG is a non-ionic galactomannan (1,6-D-Galactose) extracted from *Cyampsis tetragonoloba* seeds, which possess exceptionally high mechanical strength, enhanced barrier properties, and antimicrobial properties [31].

On the other hand, poloxamers are synthetic triblock copolymers used as solubilizing agents (stabilizer/surfactant) [32] and as pharmaceutical excipients [33] to produce gelation and coating agents for drug delivery systems [32,34]. In particular, poloxamer 188 (P_188_) has been utilized in biological formulations [35,36,37,38] for its antimicrobial activity and its ability to improve the wound closure during clinic management [39,40]. 

Our study aimed to fabricate and evaluate a biopolymeric membrane for wound healing utilizing SA, XG, and GG as a biopolymer matrix, integrated with insulin for possible drug repositioning in wound care and supplemented with P_188_ as an insulin stabilizer and antibacterial agent. The membranes were prepared employing a solvent casting method with different P_188_ concentrations (1, 5, 10, and 30% *w*/*v*) to stabilize the insulin (50 IU/mL). The characterization involved a comprehensive analysis of the physicochemical properties, mechanical strength, antioxidant capacity, and biological performance through in vitro and in vivo assays, including antibacterial efficacy, cellular viability, scratch wound healing tests, and an excisional wound model in rats.

In summary, the insulin-enriched biopolymeric membranes demonstrated several advantages for wound treatment, such as the low cost for their fabrication (due to the biopolymers used), ease of handling, robust mechanical resistance, transparency, high swelling capability (1100%), 30% antioxidant activity, and antibacterial activity (*S. aureus* 85% and *P. aeruginosa* 75% of growth inhibition, respectively). Moreover, the membranes promoted a 5.5-fold increase in cellular migration and 72% closure at 24 h, with a 3.2-fold increase and 92% in vivo closure at 10 days, essential for wound closure. Consequently, we present this material as an innovation with potential for wound healing.

## 2. Materials and Methods

### 2.1. Materials

Sodium alginate (SA) and calcium chloride (CaCl_2_) were purchased by Meyer (Ciudad de Mexico, Mexico); xanthan gum (XG), guar gum (GG), glycerol (Glic), and sorbitol (Sorb) were supplied by Cosmopolita Droguery (Ciudad de México, Mexico). Phosphate buffered saline (PBS) and P_188_ were purchased by Sigma Aldrich (KGaA, Darmstadt, Germany). Human recombinant insulin (100 IU, Aurax) was obtained by PISA laboratories (Ciudad de México, Mexico). Clindamycin (Dalatina gel 1%, MAVI Farmacéutica, Ciudad de México, Mexico) and Ciprofloxacin (Sophixin ointment 0.3%, Laboratorios Sophia, Ciudad de México, Mexico) were acquired as medicinal products, respectively. All other chemicals and reagents were of at least analytical-grade quality.

### 2.2. Methods

#### 2.2.1. Membranes Preparation

##### Preparation of Stock Gel

The membranes were fabricated with sodium alginate (3% *w*/*v*) using the solvent casting method [41]. The gel was prepared by dissolving CaCl_2_ (0.1% *w*/*v*) as a cross-linking agent in distilled water at 40 ± 2 °C. We gently dissolved the cross-linker and slowly added the solid mixture of polymers SA/XG/GG (3/1/1% *w*/*v*, respectively). Finally, the mixture of plasticizers Glic/Sorb (25/10% *w*/*w*, respectively) was incorporated by mechanical stirring. The polymer gel was reposed at 4 ± 2 °C for 24 h and cast to obtain membranes. 

##### Insulin-Poloxamer 188 Solution (IP_188_)

P_188_ solutions were prepared at 1, 5, 10, and 30% (*w*/*v*) at 4 ± 2 °C by magnetic stirring overnight and filtered (0.45 µm disk). Insulin solution (50 IU/mL) was prepared with PBS (pH 7.4), and, after, the pH was adjusted to 8.0 ± 0.20 [25]. The mixtures were prepared with 1 mL of P_188_ solution for each concentration (1, 5, 10, and 30% *w*/*v* P_188_) and 1 mL of insulin solution gently stirred for 30 min at 4 ± 2 °C.

##### Ensemble of Biopolymeric Insulin Membranes

To obtain the membranes, 15 g of polymeric gel stock was dissolved with 30 mL of distilled water to have a fluid gel; after, 2 mL of IP_188_ (30% *w*/*v*) solution was incorporated into the gel solution at 4 ± 2 °C. The final solution was poured into a Petri dish (10 cm diameter) and dried in an incubator (Ecoshel, FCA 3000 Serials, Pharr, TX, USA) at 30 ± 2 °C for 48 h. 

Additionally, we evaluated low concentrations of IP_188_ (1, 5, and 10% *w*/*v*) and the order of addition (after the solvent casting method as an absorption process by swelling) to maintain higher insulin stability [42], unlike the 30% *w*/*v* P_188_ formulation that was incorporated from the hydrogel preparation.

#### 2.2.2. Physicochemical and Mechanical Characterization

##### Fourier Transform Infrared Spectroscopy (FTIR)

We used an FTIR with UATR diamond and monitored pressure (PerkinElmer, Spectrum two, Waltham, MA, USA) from 500 cm^−1^ to 4000 cm^−1^ to evidence different chemical interactions between excipients. The spectra were recorded in absorbance, and their process was completed with the commercially available software IR (SpectrumTM 10). 

##### Homogeneity of Thickness and pH

The weight was determined (*n* = 7) with a total membrane (Ohaus, PR224ZH/E, Shangai, China), and the average thickness was evaluated utilizing a digital micrometer (Steren, HER-411, Ciudad de México, Mexico) with 0.1 mm (0.001 in) of resolution at ten randomly scattered points for each film. The results were expressed by the mean of the measurements ± standard deviation (SD). 

The pH values of gel solutions (1 cm^2^ membranes were dissolved in 3 mL of PBS medium until total dissolution) were measured through a pH meter (Worner Lab, PHS-3C, Shaoxing, China). 

##### Transparency and Transmittance

Membrane light transmission and transparency were determined by the UV–Vis spectrophotometry method (DLAB, SP-V1000, Beijing, China) [43]. Rectangular membrane samples (10 × 40 mm) were cut and placed into a spectrophotometer cell. The light barrier properties were measured at wavelengths between 200 and 800 nm, using air as a control. The equations determined the transparency and opacity of the membranes:

$$Transparency:\;\frac{\;Log\;\%T_{600}\;}{x}$$

$$Opacity:\frac{Abs_{500}}{x}$$

where *%T*_600_ is the average percent transmittance at 600 nm, *Abs*_500_ represents the average absorbance value at 500 nm, and *x* corresponds to the film thickness (mm). Three samples were used for each condition.

##### Water Content

The membranes were cut into fragments of 1 cm^2^ and weighed before and after 24 h in an oven (Ecoshel, FCA 3000 Serials, USA) at 40 °C. The weight of the fragments was monitored until a constant parameter was obtained [44]. The water content or moisture content (MC) of the films was determined with the following equation:

$$Moisture\;content:\;\frac{\;W_{2}-W_{1}}{W_{1}}\times 100$$

where *W*_2_ is the weight of the membranes after drying, and *W*_1_ is the initial weight. 

##### Contact Angle

In order to determine the membrane’s hydrophobicity, the contact angle (CA) was determined with water droplets on the film surface (*n* = 3) [45]. A glass pipette dropped 24.5 ± 0.63 μL of distilled water on the sample (1 cm^2^), and images were recorded using a digital camera. The contact angle was calculated as the angle between the tangent line on the droplet at the point of contact and the line drawn along the surface of the membrane using ImageJ software, 2019 version.

##### Porosity

We used the liquid displacement method with *n*-hexane to determine the membrane porosity [46]. Previously, the membrane sample (1 cm^2^) was dried until constant weight at 40 °C. The sample was immersed in hexane and stored at 37 °C for 30 min, and after the sample was retired of dissolvent and weighed. The equation calculated the porosity index of membranes.

$$\%\;P=\frac{\left[\frac{W_{2}-W_{1}}{\rho_{h}}\right]}{\left[\frac{W_{2}-W_{1}}{\rho_{h}}\right]+\left[\frac{W_{1}}{\rho_{h}}\right]}\times 100$$

*W*_1_ is the initial weight, *W*_2_ is the weight after treatment with hexane adsorbent, and *ρ_h_* represents the hexane density.

##### Occlusion Test and Water Vapor Transmission Rate (WVTR)

The occlusion test was carried out with minor modifications [44]. Briefly, a 15 mL flask was filled with 10 mL of PBS (pH 7.4), sealed with biopolymeric insulin membranes (15 mm diameter), and stored at 32 ± 2 °C during different time points. The occlusion factor was determined using the following equation:

$$Occlusion\;Factor=\frac{A-B}{A}\times 100$$

where *A* is a negative control (flask without membranes), and *B* is the difference in water loss of membranes. 

The rate of water vapor transmission rate (WVTR) was determined using the following equation:

$$WVTR=\frac{W_{i}-W_{f}}{A\times t}$$

where *W_i_* − *W_f_* is a water loss weight (g), *A* is the area of the exposed membrane (m^2^), and *t* is a time (24 h) [47].

##### Swelling Behavior

Fragments of 1 cm^2^ were weighed, and the samples were submerged in 3 mL of PBS (pH 7.4) and incubated at 32 ± 2 °C at different periods. After different times, the rest of the buffer was removed, and the membrane was weighed [48]. The swelling index was calculated with the following equation:

$$Swelling\;index:\;\frac{\;W_{2}-W_{1}}{W_{1}}\times 100\%$$

where *W*_1_ is the weight of the membrane before swelling, and *W*_2_ is the weight after swelling.

##### Membrane Dissolution Test 

The dissolution test was determined by the weight loss of membranes [49]. The samples (1 cm^2^) were submerged in PBS (pH 7.4) at 32 ± 2 °C with magnetic stirring of 500 rpm (DLAB, MS-M-S16, China) during different intervals of time, then were removed, dried at 40 ± 2 °C for 24 h, and weighed. The weight loss ratio was calculated according to the following equation:

$$Weight\;loss\;ratio=\frac{W_{1}-W_{2}}{W_{1}}\times 100\%$$

*W*_1_ is the initial weight, and *W*_2_ is after the dissolution test. 

##### Mechanical Properties

Mechanical properties were determined using a universal testing machine (Macmesin, Multitest1-i, Sterling, VA, USA). Membrane strips (*n* = 6) of 40 × 40 mm dimensions were individually attached to the machine probe, and a tensile load was applied at a crosshead speed of 50 mm/min until the film broke. The tensile strength, % of elongation, and Young’s modulus were calculated with the following equations:

$$TR=\frac{F_{max}}{A}\;\;\;\;\;\;\;\;\;\;\;\;\;\;\;\;\;\;\;\%\;E=\frac{L}{L_{0}}\times 100\;\;\;\;\;\;\;\;\;\;\;\;E=\frac{\sigma }{\varepsilon }$$

where *TR* is the Traction Resistance, *F_max_* is the maximum strength of the membrane at the time of breakage, and *A* is the transversal area. For % elongation (*% E*), *L*_0_ is the initial length of the membrane, and *L* is the membrane length at the time of breakage. For Young’s modulus (*E*), the *σ* and *ε* represent the tension and deformation, respectively [50].

#### 2.2.3. Antioxidant Activity (DPPH)

The 2,2-diphenyl-1-picrylhydrazil (DPPH) free radical scavenging was measured according to the method described previously by Romero-Montero, 2020 [51]. The DPPH solution was prepared in ethanol at 6.34 × 10^−5^ M. 290 µL of DPPH solution and 10 µL of each concentration sample (15 mg/mL and 0.21 IU/mL for membranes and insulin, respectively, with five serial micro-dilutions) were incubated on a 96-well plate for 30 min at room temperature. The spectroscopic method determined the DPPH radical inhibition at 517 nm (Agilent, EPOCHH-SN, Santa Clara, CA, USA). The equation estimated the relative percent of radical scavenging activity of DPPH (RSA) as follows:

$$RSA=\left|\frac{A_{s}-A_{0}}{A_{0}}\right|\times 100$$

where *A_s_* and *A*_0_ were the absorbance sample and control, respectively.

#### 2.2.4. Biological Activity

##### Antibacterial Properties

We used the microdilution method for the antibacterial activity against *Staphylococcus aureus* (ATCC 25923) and *Pseudomonas aeruginosa* (ATCC 27853). For the assay, we utilized Muller Hinton Broth (MHB), active culture (24 h of incubation) at 0.3 McFarland concentration (1 × 10^5^ CFU), and samples with 15 mg/mL for stock solution (UV sterile conditions). On a 96-well plate, we put 100 µL of MHB for each well (positive control). We added 100 µL of stock solution of each sample, mixed, and extracted 100 µL for the next column from the serial dilution to have five concentrations; for each well, add 100 µL of active culture, except for the negative control. We applied clindamycin (0.07–1.25 mg/mL, Dalatina gel 1%, MAVI Farmacéutica, Ciudad de México, Mexico) and ciprofloxacin (1.30–20.80 mg/mL, Sophixin ointment 0.3 %, Laboratorios Sophia, Zapopan, Mexico) as positive controls. The incubation was for 24 and 48 h. The concentration was determined by optic density (λ= 620 nm) (Agilent, EPOCHH-SN, USA). The study was performed in triplicate. 

##### Cell Viability Assay

BJ fibroblast cells (ATCC: CRL-2522TM) were cultured in Modified Eagle Medium (MEM, Invitrogen, Waltham, MA, USA), supplemented with 15% Fetal Bovine Serum, 1% penicillin/streptomycin, and, at 37 °C in a humidified atmosphere with 5% CO_2_. The culture medium was changed every two days to have a cell concentration of 1 × 10^5^ cells. For the viability assay, membranes were weighted (30 mg) and UV-sterilized for 30 min. Samples were solubilized with two mL of PBS medium overnight. After that, samples were homogenized with mechanical micropipette force. Solution samples were added to the cell culture, and the viability was determined after 24 h against BJ cells using 3-(4,5-dimetylthiazol-2-yl)-2,5-diphenyl-tetrazolium bromide (MTT) assay. The absorbance lectures at 450 nm correlated with the viability assay using a microplate absorbance reader (ImarkTM, BIORAD, Hercules, CA, USA). The relative viability was compared to the control viability, using three repetitions.

##### Scratch Assay In Vitro

Fibroblast BJ (ATCC: CRL-2522) was cultured on a 24-well culture plate for 24 h until a confluence of 80% was achieved. The scratch was conducted using a 1000 µL micropipette tip followed by a wash using PBS, and later treatment (15 mg/mL membrane concentration) was administered; the culture was conducted at 37 °C in serum starvation. Pictures were captured at 0, 4, 8, 12, and 24 h (Thermo Fisher Invitrogen, Evos FL 2 Cell Imagin System, Alcobendas, Spain) following the scratch, while the cells were cultured at 37 °C. The area of the scratch (%) was measured using ImageJ and the Wound Healing Size Tool plugin version 1.54j [52]. 

##### In Vivo Wound Healing Assay

The study included ten male Wistar rats weighing 250–300 g. The Institutional Committee for the Care and Use of Laboratory Animals, Faculty of Veterinary Medicine, UNAM, Mexico, approved the experimental procedures for the Care and Use of Experimental Animals in the present article (Trade number: FMVZ/168). Also, all experimental methods were designed according to Mexican legislation NOM-062-ZOO-1999. All animals were housed in polycarbonate cages with stainless steel covers in a controlled temperature room at 20 °C (12 h light/dark cycle and relative humidity of 50 ± 10%). Throughout the experiment, rats had free access to water and pellet laboratory chow BIO-DIETA-LAB 7300 (ABENE^®^, Atizapán de Zaragoza, Mexico). 

The rats were randomly divided into control, 0% *w*/*v* IP_188_, and 30% *w*/*v* IP_188_. The rats were anesthetized with ketamine (100 mg/kg i.p.) and xylazine (5 mg/kg i.p.). After anesthetization, shaving, and sterilization, two full-thickness wounds (10 mm in diameter) were meticulously created on each side of the rats’ backs to ensure consistent and controlled injuries for the study. The control group received no therapeutic intervention, while the 0% *w*/*v* IP_188_ and 30% *w*/*v* IP_188_ groups received 1.2 cm^2^ of a membrane (equivalent to 82 mg of formulation that includes 0.7 IU of insulin) immediately after the skin wounds were inflicted and at 6, 24, and 48 h following the procedure. 

Animals were housed in individual cages. Photographs of the wounds were captured on days 0 (at application), 3, 5, 7, and 10 to assess wound closure progression. This enabled comprehensive comparisons and determination of closure percentages across the three specified groups. The wound area was calculated by tracing the wound margins and evaluated as a percentage of the original wound using Image-Pro Plus 6.0 software. The wound percentages were evaluated statistically using the two-way ANOVA test using Graph Pad Prisma software^®^ version 5.0. A probability value of *p* ≤ 0.05 was considered significant.

To determine the standard glucose value in our rats, we randomly collected a blood sample from three animals one day before treatment application at 0, 15, 30, 45, 60, and 90 min (baseline group). On the day of treatment (day 0), we collected blood at 15 min before applying the treatment (−15 min), 0 min at the application of the membrane, and 15, 30, 45, and 60 min and 24 h after the first administration to evaluate the effect of topic insulin in the formulation on systemic glucose in experimental groups. 

Samples were obtained for a duplicate from coccygeal vein puncture using a glucometer (Contour Plus^®^, Ciudad de México, Mexico) and test strips (Contour Plus^®^, Ciudad de México, Mexico) in the three experimental groups. We obtained the mean each time and performed two-way ANOVA to analyze the results using Graph Pad Prisma^®^ software version 5.0. A probability value of *p* ≤ 0.05 was considered significant. 

## 3. Results and Discussion

### 3.1. Chemical Properties

The evaluation of the chemical composition of the biopolymeric insulin membranes is important to recognize the possible chemical interactions between the excipients. The membranes comprised three biopolymers (SA, XG, and GG) and two plasticizers (Glic and Sorb) that predominated in high concentrations. The FTIR spectra of SA, XG, and GG (Figure S1a, S1b, and S1c, respectively) revealed characteristic bands at 3250 cm^−1^ for the –OH groups, bands indicative of vibrations of –C=O in 1601, 1644, and 1307 cm^−1^, and –CH_2_ and –CH groups in 1000–1400 cm^−1^. The plasticizers presented FTIR bands at 3250 and 2998 cm^−1^ for the –OH and –CH vibrations, respectively (Figure S1d,e). The physical mixture (Figure S1g) and vehicle membrane (Figure S2a) at 0% *w*/*v* IP_188_ exhibited the principal bands of SA (Figure S1a). 

Figure S2b–e represent the membrane spectra with different concentrations of IP_188_ (1, 5, 10, and 30% *w*/*v*, respectively) and revealed the bands of the –OH, –CH_2_, and –CH groups’ characteristics of P_188_. The –CO vibrations appeared different; a short and wide signal in the ranges 1602–1647 and 1412–1460 cm^−1^ was for the primary and tertiary amides of insulin, respectively [53,54], evidencing the integrity of the excipients. The insulin maintained stability during solvent-casting and was stored at 4 °C for one month (Figure S3), the maximum desired time of possible application in a chronic wound.

### 3.2. Physical Characterization (Appearance, Weight, Thickness Homogeneity, and pH)

**Figure 1:**
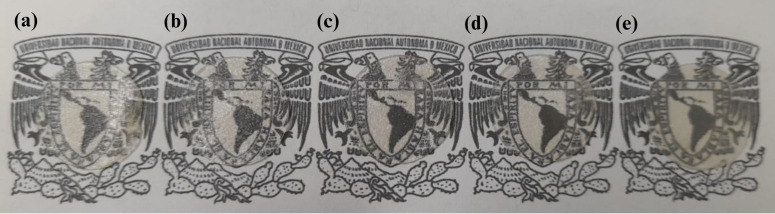
Physical membranes’ appearance of biopolymeric insulin membranes. (**a**) Vehicle membrane (0% *w*/*v* IP_188_); insulin membranes with different poloxamer 188 concentrations: (**b**) 1% *w*/*v* IP_188_; (**c**) 5% *w*/*v* IP_188_; (**d**) 10% *w*/*v* IP_188_; and (**e**) 30% *w*/*v* IP_188_.

After drying, all the membranes were homogeneous and transparent, presenting a smooth surface (Figure 1), flexibility, and adherence properties. The membranes’ weights ranged from 3.36 ± 0.14 to 3.54 ± 0.06 g per piece, and the thickness for the 0, 1, 5, 10, and 30% *w*/*v* IP_188_ membranes varied from 410 ± 0.05 to 560 ± 0.06 µm (Table 1). 

The biopolymeric membranes without insulin and poloxamer 188 (0% *w*/*v* IP_188_) were transparent, while the insulin-poloxamer hydrogel-loaded membranes (30% *w*/*v* IP_188_) and the addition after the solvent casting method (1, 5, and 10% *w*/*v* IP_188_) by a process of absorption through swelling became translucent in appearance. Increasing the amount of poloxamer 188 increases the conversion from transparent to translucent, but with the ease of distinguishing the possible wound bed [55], as depicted in Figure 1. The membranes were homogenous systems, correlated with the thickness (410 ± 0.05–560 ± 0.06 µm) and insulin-poloxamer concentrations (1–30 *w*/*v* IP_188_), supported in the standardization of the solvent-casting manufacturing method, mainly controlling the drying temperature [56] at 48 h/32 °C. An appropriate thickness represents a comfortable administration; the values for an anatomic permanent dispositive over a wound are in thickness ranges of 260–840 µm, associated with the dermis thickness of 500–2000 µm [56]. The thickness values of the biopolymeric insulin membranes (0–30 *w*/*v* IP_188_) are similar compared with the results of other biodegradable alginate membranes, such as sodium alginate–hyaluronic acid with 321 ± 0.01–450 ± 0.20 µm [47] and sodium alginate–chitosan–silk fibroin of 510 ± 0.09 µm [57]. 

Wound healing is a complex process influenced by intrinsic and extrinsic factors like wound pH. The skin pH of chronic wounds is between 7.2 and 8.9 [48]; in most cases, an infection with a basic pH for bacteria ammonia production accompanies chronic wounds. Then, a wound bed equilibrium, decreasing pH (pH 3.2–5.4) with a wound dressing [58], could help to present antimicrobial effectiveness and fibroblast proliferation [59]. Our membranes presented a slightly acidic pH in the 5.80 ± 0.07–6.24 ± 0.07 range, and these values are compared and correspond with other biopolymeric wound dressings such as sodium alginate–curcumin nanoparticle (SA-CurNp) membranes with pH ranges of 5.65 ± 0.01–5.97 ± 0.05 [48] and sodium alginate–cannabidiol and cannabigerol nanoparticle films with pH values of 6–7 [59]. 

### 3.3. Light Barrier Properties, Transparency, and Opacity

**Figure 2:**
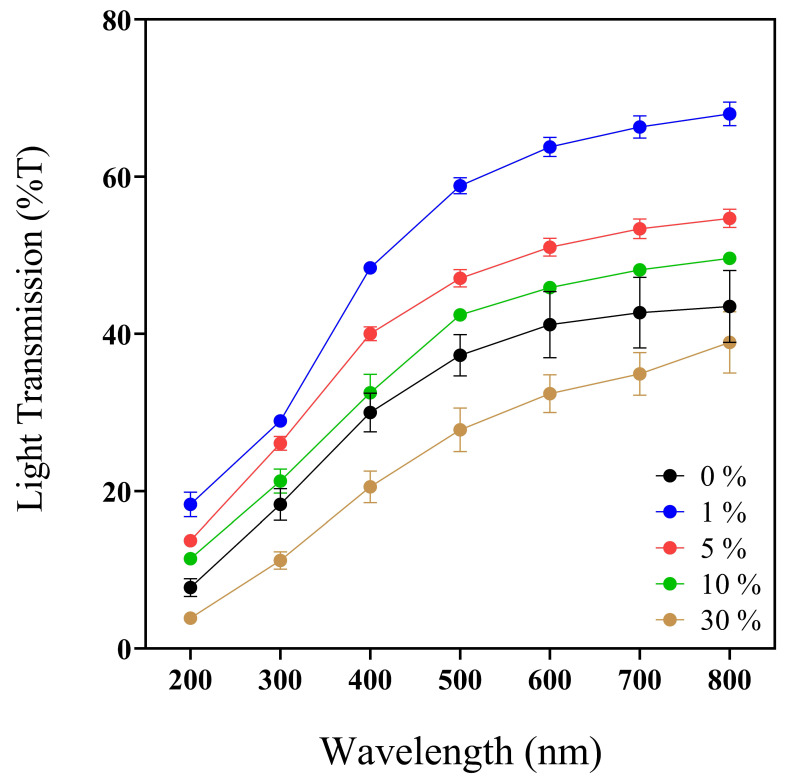
UV–Vis spectra profile (200–800 nm) of 0, 1, 5, 10, and 30% *w*/*v* IP_188_ membranes. Transmission values expressed in mean ± SD, *n* = 3.

Wounds are susceptible to UV radiation; even skin that has closed the wound is susceptible to UV [60]. Then, the optical properties of biomaterials are essential to determine UV light protection (200–400 nm) (Figure 2), transparency and opacity factors (400–800 nm), and prominent values to facilitate wound monitoring without membrane removal [61]. 

Previous works reported the convenient UV-barrier properties of different biopolymers such as chitosan–polyvinyl alcohol with *Mentha spicata* L. and *Punica granatum* ranging between 0 and 12.5% T [62] and calcium–alginate–*Aloe vera* 0–25% films with UV values ranging between 37 and 75% T [61]. Analyzing the UV light protection capacity of our biopolymeric insulin membranes (0–30% *w*/*v* IP_188_) (Figure 2, Table 2), the values ranged from 3.84 ± 0.65 to 18.32 ± 2.73% at T_200nm_ and 20.53 ± 3.46 to 48.41 ± 1.25% at T_400nm_. The values indicated that the biopolymeric insulin membranes had lower UV protection properties than the chitosan–polyvinyl alcohol films but similar UV values to the calcium–alginate–*Aloe vera* films. However, it depends on the UV analyzed range. 

Regarding the transparency and opacity values of the 0–30% *w*/*v* IP_188_ membranes, we registered a range of 3.09 ± 0.10–4.17 ± 0.02 and 0.38 ± 0.02–0.98 ± 0.10, respectively. Our biopolymeric insulin membranes presented intermedium transparency properties compared to the results of another biodegradable film. As demonstrated in Figure 1, it enables visual appreciation through the membrane, which could facilitate the medical monitoring of the injury. The transparency and opacity properties of our membrane are following other previously investigated materials such as chitosan–polyvinyl alcohol films with *Mentha spicata* L. and *Punica granatum* (transparence/opacity values ranged between 6.71 and 9.13/0.08 and 0.17, respectively) [62] and calcium–alginate–*Aloe vera* (transparence value 1.28–2.11) [61]. 

### 3.4. Physical and Barrier Properties of the Membranes (Water Content, Contact Angle, Porosity, Occlusion, Swelling, and Dissolution)

The water/moisture content (MC) (Table 3) is the parameter that describes the amount of water molecules occupying the empty volume of the microstructure of the membrane [63]. Also, water molecules act as a plasticizer in the membrane matrix, especially in the case of hydrophilic biopolymers [64]. 

The results represented a short range of MC (8.84 ± 0.71–13.60 ± 1.70%/cm^2^) of the biopolymeric insulin membranes after the solvent-casting process, which was controlled with a low temperature of drying (30 ± 2 °C). Some films with low moisture content are arrowroot starch films (8.84–13.3% MC) [65] and eggshell/orange enriched with banana starch bio-absorbent films (9.98 ± 0.46–11.23 ±0.72% MC) [66]. The low moisture content demonstrated beneficial physical characteristics such as mechanical properties and prevented microorganism growth for storage life [44,67]. 

The contact angle on the membrane surface determines the hydrophobicity or wettability of the wound dressing. Values > 150° CA described superhydrophobic surfaces, while, if CA is <90°, the surface is called hydrophilic material [68]. The value depends on the relation between the liquid–solid adhesive or cohesive forces [69]. 

When we added insulin and P_188_ directly at the beginning of the solvent casting method (30% *w*/*v* IP_188_) during gel preparation, the contact angle of the membrane increased from 21.48 ± 1.43° to 25.04 ± 1.59°. The 1, 5, and 10% *w*/*v* IP_188_ membranes presented CA values of Ɵ = 28.82 ± 1.24°, 35.29 ± 1.42°, and 38.23 ± 1.71°, respectively, higher than the 0% *w*/*v* IP_188_ membranes (Ɵ = 21.48 ± 1.43°). The increase in values is due to the low porosity index (Table 3) [70] and the poloxamer content (1, 5, and 10% *w*/*v*). The CA value of poloxamer 188 is Ɵ = 56.8 ± 1.8° [71,72]. Thus, the contact angles for the 1, 5, and 10% *w*/*v* IP_188_ increased [61] proportionally to the P_188_ content (Table 3), possibly due to superficial exposure to poloxamer. The 30% *w*/*v* IP_188_ membranes presented a CA value of Ɵ = 25.04 ± 1.59°, lower than the 1, 5, and 10% *w*/*v* IP_188_ membranes. The difference in CA values is because the formulations of the 1, 5, and 10% *w*/*v* IP_188_ membranes incorporate poloxamer after the solvent-casting through absorption due to swelling, while the 30% *w*/*v* IP_188_ membranes incorporate it at the beginning of the solvent-casting process, even when these membranes presented high poloxamer concentrations. The values were accorded with a hydrophilic surface [68] of biodegradable natural polymers such as chitosan film-loaded CuS nanoparticles with polyphenols extracts (Ɵ = 36.9°–53.7°) [73] and alginate reticulated with Ca^2+^ (Ɵ = 42.2°) and loaded with 25% (*w*/*v*) *Aloe vera* films (Ɵ = 30.3°) [61]. 

The porosity index of membranes is an important feature in evaluating wound healing properties, related to the adhesion and proliferation process allowing cells to penetrate through the porous membrane and the diffusion of nutrients [42,74,75]. The porosity also determines the angle contact, moisture content, vapor transmission, and mechanical properties [43]. This study investigated porosity on dry membranes, and we used hexane as a displacement dissolvent method [46]. 

The solution of IP_188_ (1, 5, and 10% *w*/*v* IP_188_) put over the 0% *w*/*v* IP_188_ membranes (with an initial porosity of 13.64 ± 1.35%) considerably decreased the porosity by forming a possible type of coating on the surface and obstructing the architecture of the initial pores. With an increase in the concentration of poloxamer (from 1 to 10% *w*/*v* IP_188_) through absorption by swelling on the membrane, a more significant decrease in the porosity was observed, with values from 1.39 to 3.95%. Meanwhile, the incorporation of IP_188_ from the beginning of the solvent-casting process (30% *w*/*v* IP_188_), not added by absorption through swelling, produced a change in the porosity towards 7.5%, so it could preserve an architecture close to the original structure since it does not form a coating film. Then, the difference in the porosities (*p* < 0.0001) was attributed to the fabrication method. The porosity values for our membranes are lower compared with other wound dressings with natural polymers such as acetate chitosan film (80%) and chitosan formate film (96%) [76]. However, the low porosity of membranes acts as a barrier to preventing microorganism colonization [56], maintaining a moist environment in the wound bed, and preventing dehydration [42]. 

One of the skin’s most critical functions is protecting our body from xenobiotics. The barrier limits topical drug permeation through the skin [44]. Occlusion or water vapor transmission is a permanent property of wound dressing materials. The permeability of water vapor across the biopolymeric insulin membrane is an essential property of cell metabolism for energy production and reparative processes like cell proliferation, re-epithelization, and collagen deposition [42,75].

**Figure 3:**
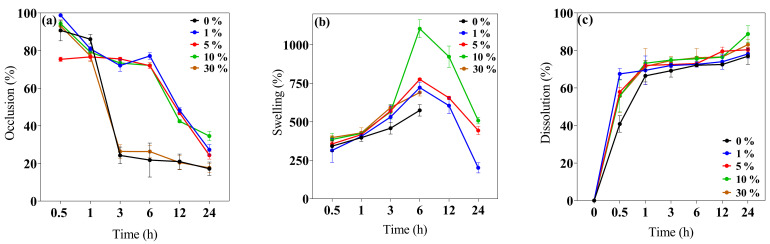
Biopolymeric insulin membrane’s physical, permanence, and barrier properties. (**a**) Occlusion, (**b**) swelling behavior, and (**c**) dissolution. Values expressed in mean ± SD (*n* = 3).

This study evaluated the 0–30% *w*/*v* IP_188_ membrane occlusion for the in vitro permeation at different periods. The results (Figure 3a) indicated that the 1, 5, and 10% *w*/*v* IP_188_ membranes presented higher occlusive factors (72.05 ± 3.06%, 75.57 ± 0.96%, and 73.38 ± 1.04%, respectively) than the 0 and 30% *w*/*v* IP_188_ membranes and were decreased regarding time; this effect was related to the porosity index, less porosity, and increased occlusion. On the other hand, the materials with a 21.05 ± 5.26% and 22.70 ± 3.22% occlusion were constant regarding time (3–24 h). In our formulations (1, 5, and 10% *w*/*v* IP_188_ membranes), as the pores were coated, the lower the porosity, the higher the occlusive factor, the higher the contact angle, and the lower the water vapor transmission rate. The water vapor transmission rate (WVTR) was estimated using the water loss method until 24 h after the evaluation (Table 3).

The wound dressing controls the WVTR for adequate wound healing. For a wound dressing to perform efficiently, the WVTR should be higher than uninjured skin and lower than injured skin. Healthy skin has 204 g/m^2^/day, first-degree burns 278 g/m^2^/day, and granular lesions 5138 g/m^2^/day WVTR values [56]. Different polymeric wound dressings presented WVTR values inside the range of granular lesions like gellan gum films (25% occlusion factor) and gellan gum–silibinin np-films (10% occlusion factor) [44], sodium alginate films (2518.2 ± 82.9–2881.1 ± 160.1 g/m^2^/day WPTR) [50], hyaluronic acid–alginate membranes (1931 and 2350 g/m^2^/day WVTR) [47], chitosan–polyvinyl alcohol films (2500–2700 g/m^2^/day WVTR) [62], and polyurethane membranes (1000–4000 g/m^2^/day WVTR) [75]. The WVTR values of the 0 and 30% *w*/*v* IP_188_ membranes were 1102 ± 19.76 g/m^2^/day and 1025.75 ± 18.25 g/m^2^/day and decreased for the 1, 5, and 10% *w*/*v* IP_188_ membranes. The values of the 0 and 30% *w*/*v* IP_188_ membranes should be used for a granular lesion dressing and the 1, 5, and 10% *w*/*v* IP_188_ membranes for a first-degree burn.

The swelling index is the capability of the wound dressing to absorb the excess exudate from the wound bed according to its morphology, which should enable the diffusion of nutrients, medium, or bioactive molecules to the cells [42,56].

The swelling behavior and dissolution index (Figure 3b,c) were investigated at 32 ± 2 °C at different periods (0.5, 1, 3, 6, 12, and 24 h). The swelling mechanism is based on the polymer chain relaxation after immersion in an aqueous medium, which results in the exposure of hydrophilic groups following the network expansion [44]. After 6 h in contact with the buffer, the 0 and 30% *w*/*v* IP_188_ membranes presented a swelling index of 573.82 ± 36.60% and 689.89 ± 22.81%, respectively. Thus, the swelling expansion increased when the IP_188_ solutions were added to the membranes. The increase in swelling of the 30% *w*/*v* IP_188_ is due to the poloxamer 188 inside the membrane and a possible increase in the hydrogen bond interactions of the poly(ethylene oxide) and poly(propylene oxide) units. However, the 1, 5, and 10% *w*/*v* IP_188_ membranes presented higher swelling ratios (721.92 ± 34.81%, 774.90 ± 3.36%, and 1104.23 ± 58.40%, respectively) than the 0 and 30% *w*/*v* membranes. Although the 30% *w*/*v* IP_188_ membranes exhibited a higher poloxamer concentration, poloxamer 188 was dispersed as a solution at the beginning of the solvent-casting method, partially hiding its interactions towards swelling due to being dispersed in the matrix of the other polymers. However, in the other formulations (1, 5, and 10% *w*/*v* IP_188_ membranes), poloxamer 188 interacts apparently from the surface with the matrix polymers, facilitating its interaction with the water molecules and, therefore, the swelling. For example, the 10% *w*/*v* IP_188_ membrane represented a double swelling index compared to the vehicle membrane. The water uptake ability was also related to the contact angle and the poloxamer concentration on the membrane surface; the greater the contact angle, the greater the swelling capacity. The observation was related to the chemical poloxamer structure and the high hydrophilic units (152 ethylene oxide units) and low hydrophobic units (29 propylene oxide units) [72]. The ideal wound dressing must also have a high water uptake capacity with 31–46% weight gained or 100–900% of water absorption [42]. The insulin biopolymeric membranes presented a percent of swelling of 573.82 ± 36.60–1104.23 ± 58.40% at 6 h; other alginate wound dressings exhibited high water uptake ability of 200–750%, 180 min [48], 200% and 1400%, 180 min [50], 150% and 400%, 80 min [65] 209.99 ± 69.24–695.94 ± 101.37% [77], and biopolymers as gelatin 535.6 ± 14.8–1059.1 ± 22.7%, 24 h [78], and chitosan–PVA 350–400% [73].

Recently, wound dressings have become attractive because of their good biocompatibility and biodegradability for decreasing plastic consumption, degrading to low-molecular-weight compounds, and absorption on the wound bed [28,79]. The in vitro dissolution profile (Figure 3c) presented a 76.94 ± 4.34–83.11 ± 2.67% of dissolution at 24 h for all the membranes. The low Ca^2+^ cross-linked enabled a quick dissolution on the medium. 

### 3.5. Mechanical Properties

**Figure 4:**
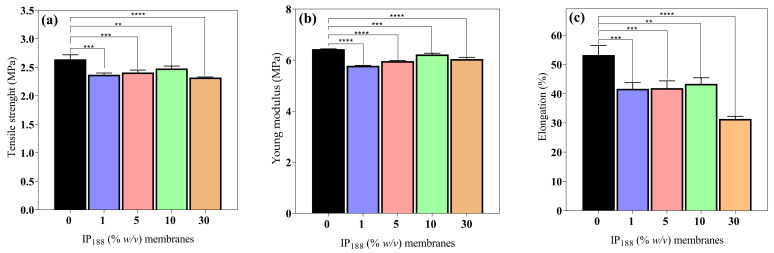
Mechanical properties of biopolymeric insulin membranes. (**a**) Tensile strength, (**b**) Young’s modulus, and (**c**) % elongation. Results are represented as the mean ± SD (*n* = 3). ** Statistically significant when compared with 0% *w*/*v* IP_188_ membrane (*p* < 0.01). *** Statistically significant when compared with 0% *w*/*v* IP_188_ membrane (*p* < 0.001). **** Statistically significant when compared with 0% *w*/*v* IP_188_ membrane (*p* < 0.0001).

Wound dressings (films/membranes) must be resistant and flexible for appropriate application, handling, and storage life [56]. The mechanical attributes (*σ*_max_, *E*, and *δ*) of the 0, 1, 5, 10, and 30% *w*/*v* IP_188_ membranes are presented in Figure 4. The mechanical values are similar between the membrane formulations. The maximum stress that membranes withstand being stretched before cracking (*σ*_max_) and the slope on the stress–strain on the elastic region (*E*) were 2.48 ± 0.04–3.32 ± 0.01 MPa and 5.78 ± 0.01–6.43 ± 0.01 MPa, respectively. The observations correspond to the recommended values (2.5–16 MPa and 4.6–20 MPa for *σ*_max_ and *E*, respectively) for wound dressings [42,56]. However, the elasticity parameter or percent of elongation (*δ*) demonstrates increased differences between the formulations. The vehicle membrane (0% *w*/*v* IP_188_) presented 53.18 ± 3.29% elongation. When we added the insulin-poloxamer solution to the membrane surface, the values decreased to 41.66 ± 2.09–43.34 ± 2.08% (1, 5, and 10% *w*/*v* IP_188_ membranes) and a lower value for the 30% *w*/*v* IP_188_ membrane (31.36 ± 0.87%) due to the chemical properties of poloxamer 188. It is possible that, in the formulations of 1, 5, and 10% *w*/*v* IP_188_ membranes, the presence of poloxamer decreases the interchain interactions and, therefore, elongation. When more significant interaction of the poloxamer with the membrane is encouraged (30% *w*/*v* IP_188_), the elongation decreases even further. The emulsifiers have been demonstrated to decrease the mechanical properties [56,73]. Borbolla et al. [56] suggested that an excellent maximum strain value (δ) is about 70%. 

However, the literature suggests that dressings with reasonable deformation and low tensile strength and Young´s modulus values have a high potential for use in medical applications such as hyaluronic acid–alginate membranes (8.15 ± 0.20 MPa, 7.52 ± 0.27 MPa and 35 ± 3.02% of *σ*_max_, *E*, and *δ*, respectively) [47], in addition to other materials such as silk fibroin–chitosan–alginate films (3.05 ± 0.41 MPa, 20.73 ± 4.6 MPa, and 45.14 ± 6.23% *σ*_max_, *E*, and *δ*, respectively) [57], alginate–PVA membranes (1.2–2.8 MPa, and 70–130%, *σ*_max_, and *δ*, respectively), and alginate–calcium membranes (5–6 MPa, 42.36–50.91 MPa, 7.86–13.56%, and 30.19–46.66% *σ*_max_, and *δ*, respectively) [61]. 

### 3.6. Antioxidant Activity

**Figure 5:**
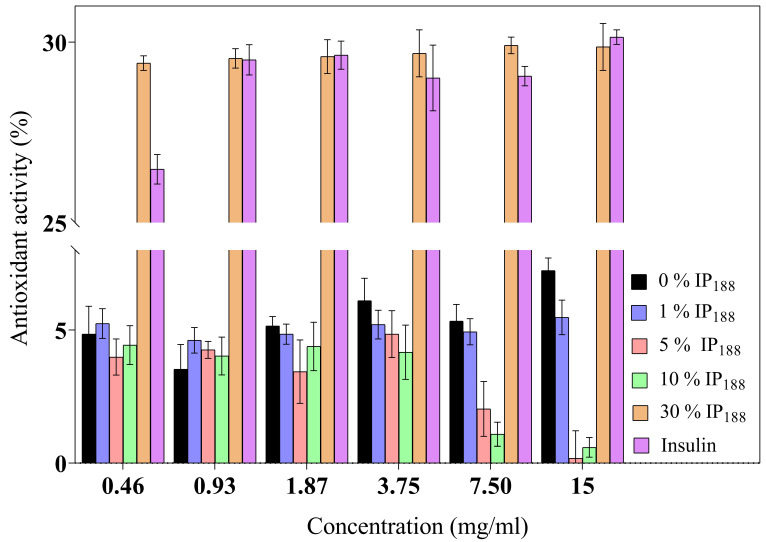
Antioxidant activity of biopolymeric insulin membranes at six membrane concentrations (0.46–15 mg/mL). Results are represented as the mean ± SD (*n* = 3). The insulin concentration remained constant, and only the poloxamer concentration varied.

The inflammatory process is a hallmark in non-healing wounds (chronic wounds); reactive oxygen species (ROS) levels produce inflammatory cells accumulated inside the wound, reflect toxicity by severe endothelial cell damage, and induce proinflammatory cytokines [8]. ROSs produce oxidative stress like hydrogen peroxide (H_2_O_2_), superanion (O^−^_2_), or peroxide (O^2−^_2_) with high values of 500 µM. In contrast, low levels of H_2_O_2_ are observed in the wound healing process during proliferation and angiogenesis [80]. Insulin is a protein that previously demonstrated a decrease in the levels of the reactive oxygen species [10] protecting lipids, proteins, and DNA. Figure 5 presents the antioxidant activity. The commercial insulin product we evaluated produced a moderate activity of free insulin (26.48 ± 0.41–30.14 ± 0.20%) for 0.01–0.21 IU/mL.

The 30% *w*/*v* IP_188_ membranes revealed a similar antioxidant activity (29.42 ± 0.20–29.87 ± 0.65%). The integrity of insulin preserves its antioxidant activity due to 30% *w*/*v* poloxamer 188 in the formulation and the integration into the membrane. The poloxamer 188 and insulin-poloxamer 188 solutions do not present antioxidant activity (0.90 ± 0.70–1.22 ± 2.12% and 1.08 ± 0.78–3.21 ± 0.90%, respectively). It is possible that poloxamer 188 interacts intimately in solution with insulin in its stabilization and hides its activity monitored in protein-alone assays. Regarding our results, previous studies demonstrated alginate sponge insulin-PLGA microparticles with decreasing H_2_O_2_ levels in an in vivo burn wound model [81].

### 3.7. Cytotoxic Assay

**Figure 6:**
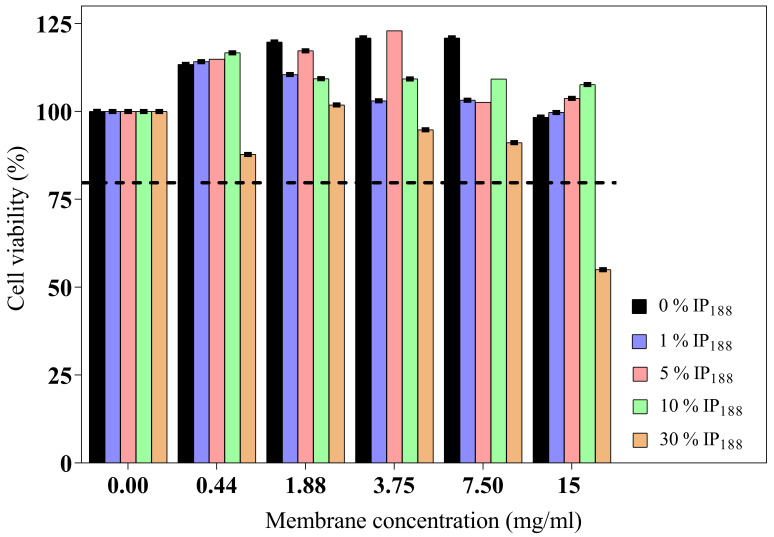
BJ fibroblast viability of biopolymeric insulin membranes. The viability test was determined on five concentrations of membranes (0.44–15 mg/mL) with different poloxamer concentrations (0, 1, 5, 10, and 30% *w*/*v* IP_188_). The dotted line indicates the minimum permitted viability. Values expressed in mean ± SD (*n* = 3).

The wound dressing should present adequate cytocompatibility for skin applications, mainly in fibroblast or keratinocyte cells [50]. The cytotoxic impact of six different concentrations (0.44–15 mg/mL) of each membrane (0–30% *w*/*v* IP_188_) on BJ fibroblasts is illustrated in Figure 6.

The wound dressing incubation for 24 h on different concentrations (0.44–15 mg/mL of membrane) indicated adequate biocompatibility and proliferation. The high poloxamer concentration (30% *w*/*v* IP_188_ membrane) promoted fibroblast reduction viability. Only a high concentration of the membrane (15 mg/mL) with the higher poloxamer concentration (30% *w*/*v* IP_188_) produced moderate cytotoxicity (55.60%) according to ISO 10993-5 [82]. The poloxamer 188 formulations had been utilized for transdermal administrations. Even low concentrations of poloxamer 188 can alter the lipid bilayer of cell membranes and weaken their structure due to a high HLB 29 [71,83]. For this, the high concentrations are not favorable for skin cell viability.

### 3.8. Antibacterial Activity

Wound infections are caused by microbial invasion, in which bacteria destroy the keratin layer of the skin and provoke dysregulation of the cellular process, cellular stress, and inflammation. The inflammatory phase of an infected wound involves many bacterial metabolisms that increase the adhesion molecules, the release of bacterial peptides and proinflammatory mediators, and increase the levels of ROS and proteases [84]. The skin pathogens in chronic wounds are commonly *S. aureus*, *P. aeruginosa*, *E. faecalis*, and *P. mirabilis*. The permanence of bacteria depends on the antibiotic treatment and the resistance mechanism of each type of bacteria [2,3]. *S. aureus* is a Gram-positive bacteria reported to have a 78.2% prevalence in chronic wounds; the principal virulence factor is the biofilm, which adheres to the wound surface [85]. *P. aeruginosa* is a Gram-negative bacteria, more common (92%) than *S. aureus*, and is predominantly a non-fermenting aerobic bacteria in chronic and burn wounds [86]. The high impact on clinical wounds required new antibacterial therapies.

We evaluated the bacterial growth inhibition (Table 4) by 0–30% *w*/*v* IP_188_ membranes and excipients as controls using the microdilution method (1.88–15 mg/mL) in two intervals of time (24 and 48 h) against two common pathogens, *S. aureus* (Figure S4) and *P. aeruginosa* (Figure S5). Table 4 presents the inhibition of *S. aureus* and *P. aeruginosa* twice (24 and 48 h). 

The inhibition of *S. aureus* (Gram-positive) was observed (Figure S4) principally with 30% *w*/*v* IP_188_ membranes (84.30 ± 2.40%) at 24 h and decreased by 20% (65.70 ± 2.10%) after 48 h passed. Our study presented membranes with moderate and low inhibition (62.70 ± 1.60%, and 65.00 ± 3.40% for 5 and 10% *w*/*v* IP_188_ membranes; 34.70 ± 2.30%, and 0% for 0 and 1% *w*/*v* IP_188_ membranes), respectively. The antibacterial activity of the membranes against *S. aureus* is mainly attributed to the poloxamer 188. This polymer produced in the formulations an activity of 64.11 ± 2.42% and 41.00 ± 3.40% inhibition for 24 and 48 h, respectively. Poloxamer 188 is a surfactant incorporated in multiple care products, has antibacterial properties, and aids wound healing. Using surfactants on wound dressing formulations has reduced the biofilm adhesion and biofilm concentration on the wound bed [39]. Table 4 and Figure S4 evidenced that insulin presented low but representative inhibition of *S. aureus* (33.70 ± 5.3%, 24 h). Previous research reported the inhibition of *S. aureus* by insulin treatment [83,87]. The inhibition of *S. aureus* by the vehicle membrane (0% *w*/*v* IP_188_) is attributed to the contribution of each of the excipients (Figure S4), such as SA (29.93 ± 1.22%, and 80.03 ± 2.0%, for 24, and 48 h, respectively), XG (57.97 ± 2.30%, and 47.31 ± 0.83%, for 24, and 48 h, respectively), GG (44.46 ± 1.48%, and 14.96 ± 6.49%, for 24, and 48 h, respectively), sorbitol (46.03 ± 0.70%, and 47.47 ± 2.29%, for 24, and 48 h, respectively), and CaCl_2_ (35.90 ± 1.76%, and 20.71 ± 2.11%, for 24, and 48 h, respectively).

Concerning the inhibition of *P. aeruginosa* (Gram-negative) (Figure S5), the low poloxamer concentration membranes (1% *w*/*v* IP_188_) presented high inhibition (74. 17 ± 0.33%, 48 h) and contrast with the high poloxamer concentration membrane (30% *w*/*v* IP_188_) because they decrease to 18.28 ± 3.70 and 12.03 ± 7.03% for 24 and 48 h, respectively. The poloxamer 188 inhibited *S. aureus* in 64.11 ± 2.42% and 41.00 ± 3.40% for 24 and 48 h, respectively. At the same time, the inhibition of *P. aeruginosa* was 49.8 ± 1.09% and 18.45 ± 1.71% for 24 and 48 h, respectively. The hydrophobic region of the poloxamer can intercalate into the bacteria’s cell wall, destabilizing the membrane and increasing the permeability. In addition, the surfactants’ properties can change the properties of the environment that surrounds them, confusing their defense mechanisms [39,88]. The insulin inhibited the *S. aureus* at 33.70 ± 5.3% and 19.3 ± 1.6% for 24 and 48 h. In comparison, *P. aeruginosa* was inhibited at 37.82 ± 2.05% and 57.08 ± 3.25% for 24 and 48 h by the presence of insulin. Figure S5 demonstrated that *P. aeruginosa* had lower inhibition than *S. aureus* by each polymer membrane component. The result could be attributed to a different type of growth metabolism (pentose via metabolism for *S. aureus* [89] and sources when supplied with nitrate as the terminal electron acceptor for *P. aeruginosa* [90]).

We did not observe an additive or synergistic effect between the membrane components; however, the activity of poloxamer 188 stands out as a predominant inhibition activity. It is possible that cross-linking of SA with CaCl_2_ traps and hinders the antibacterial activity of each component. Regarding the clinical application, the 30% *w*/*v* IP_188_ membrane inhibition values of 84% against *S. aureus* and 50% against *P. aeruginosa* stand out prominently because they are ingredients that do not contain a formal antibiotic. Our results showed a similar activity to that observed with the commercial pharmaceutical preparation of clindamycin.

### 3.9. Scratch Assay

**Figure 7:**
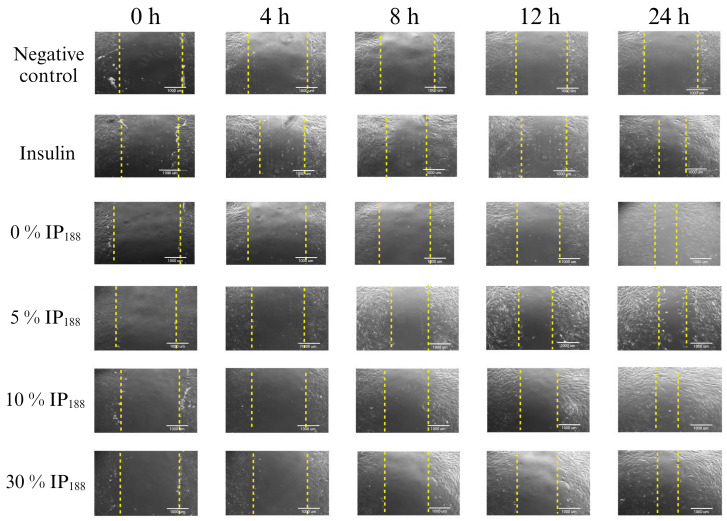
Scratch wound healing assay. Time-lapse of insulin biopolymeric membranes’ in vitro wound healing process (0, 1, 5, 10, and 30% *w*/*v* IP_188_). The study was carried out at a membrane concentration of 15 mg/mL. The dotted lines indicate the delimitation of the progressive closure of the lesion.

**Figure 8:**
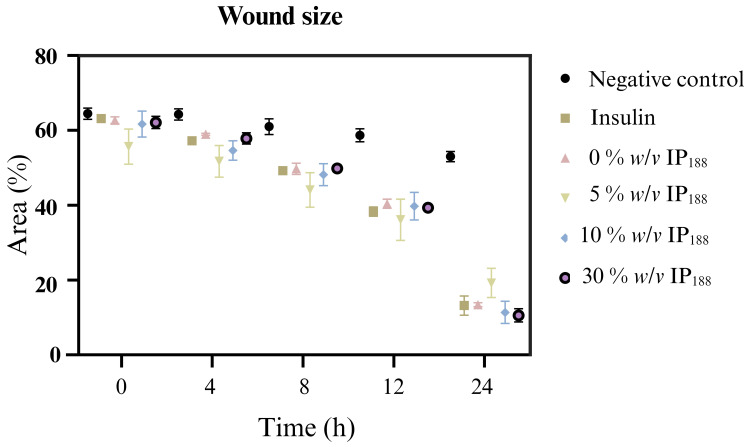
Relative wound area calculated by the in vitro scratch assay of biopolymeric insulin membranes (0, 1, 5, 10, and 30% *w*/*v* IP_188_). The study was carried out at a membrane concentration of 15 mg/mL.

Migration is a process involved in the phases of wound healing; it is the ability of adhesion cells to move from the extreme to the center of the wounds to generate granulation tissue and posterior wound closure [8]. Figure 7 presents the healing process at the lapse of time, and Figure 8 illustrates the graphic of the relative wound area (%) calculated by the distance between the cells migrated at each time. 

The in vitro wound healing assay with polymeric insulin membranes (0, 5–30% IP_188_) and free insulin registered a similar decrease in the area of the incision with 55.07 ± 5.11 to 72.34 ± 6.05% and 75.18 ± 3.87%, respectively, for 24 h of treatment. Insulin is a peptide hormone that regulates blood sugar levels; previous in vitro [14], in vivo [10,11], and clinical [11] assays demonstrated the closure of wounds with delivery systems as microparticles [14], nanoparticles [91,92], and gel/hydrogels [24,93]. In this work, the polymeric insulin membranes demonstrated convenient in vitro migration, confirming the preservation of the therapeutic protein since insulin can be a biological drug repositioning for wound healing.

### 3.10. In Vivo Assay

**Figure 9:**
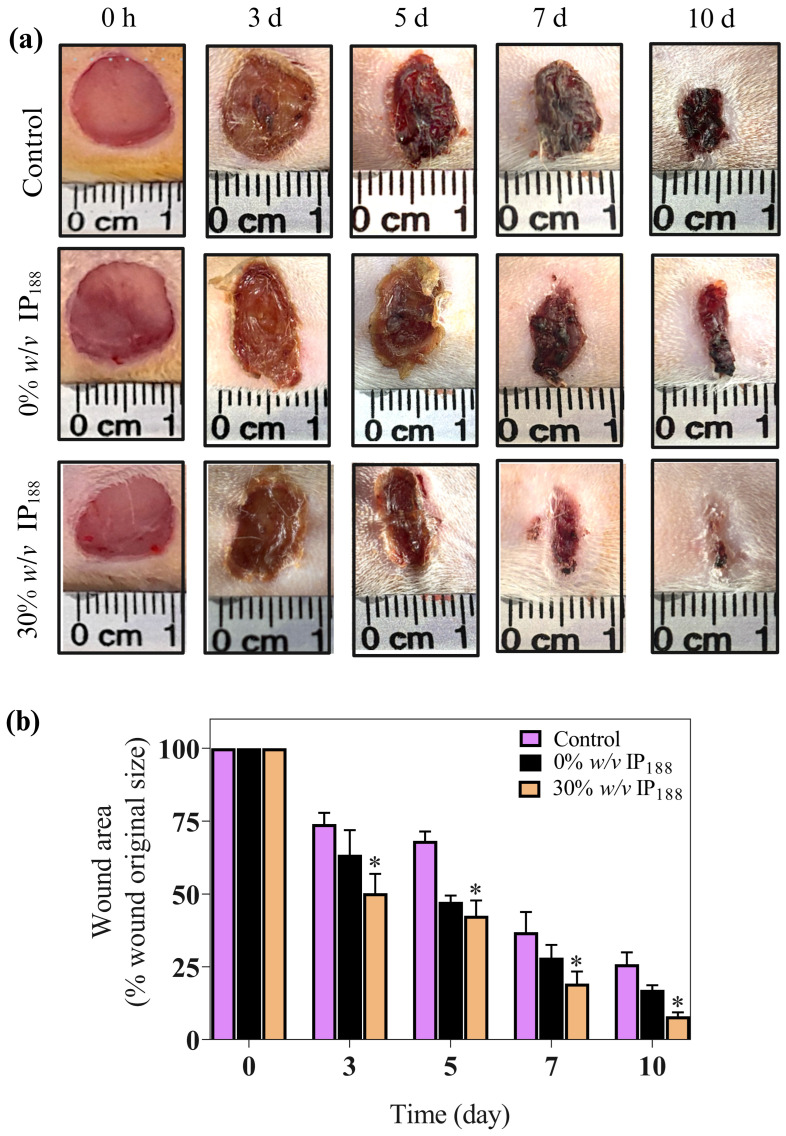
Insulin accelerates excisional wound healing in male Wistar rats. This study used the wound healing model. Wistar rats weighing 250–300 g were anesthetized, shaved, and prepared to produce the injury. Two full-thickness wounds (10 mm in diameter) were created on each side of the rats’ backs. We applied 1.2 cm^2^ of a membrane (equivalent to 82 mg of formulation that includes 0.7 IU of insulin) in 0% *w*/*v* IP_188_ and 30% *w*/*v* IP_188_ groups immediately after the skin wounds were produced. (**a**) Representative images of the healing process on 3, 5, 7, and 10 days; (**b**) wound healing rates at different times. Healing rates of full-thickness cutaneous wounds were significantly increased in the group 30% *w*/*v* IP_188_ at 3, 5, 7, and 10 days * *p* < 0.05 versus the control group.

In our in vitro results, insulin promoted wound closure with a 5.5-fold increase and 72% closure at 24 h compared with the groups without insulin. Therefore, the wound healing effects of the insulin were investigated in full-thickness cutaneous wounds in male Wistar rats. All the animals exhibited no significant side effects during the experiments, such as mortality or signs of disease. As depicted in Figure 9, the wounds treated with insulin (30% *w*/*v* IP_188_ group) healed faster than the controls (Figure 9a). 

We observed an important improvement in the healing appearance 24 h after membrane application and significant results in wound healing since day 3 in the 30% *w*/*v* IP_188_ group compared to the controls (*p* < 0.05) (Figure 9b). This trend continued, with the 30% *w*/*v* IP_188_ group consistently showing the most significant reduction in wound area through days 5, 7, and 10, indicating superior wound healing. Therefore, as other studies have shown, our membrane demonstrates high efficacy. For example, a study by Apikoglu-Rabus (2010) [94] used 20 μL of regular human insulin mixed with sterilized water in rats twice a day until the end of the experiment (day 15). The authors reported that the complete epithelization time was an average of 8 days, and by day 3 the wound healing was already notable. Negrini et al. (2017) [95] administered porcine insulin at 5 IU/mL diluted in glycerol topically 6 h after wound induction and daily during the first week following an injury. The researchers also observed significant differences on day 28 in the wound contraction in the insulin-treated group. A significant difference in our research is that we only applied the membrane in the first two days after the wound. The membrane, probably being in the wound for longer, had a prolonged effect compared to applying water with insulin.

Additionally, the dose that we used (0.7 IU) is like the one reported as the best for promoting wound healing in the study by Lima (2012) [96] that evaluated the effectiveness of different concentrations of insulin in cream form (0.0, 0.1, 0.25, 0.5, and 1.0 IU/100 g) on wound healing. The authors observed that the concentrations of 0.5 IU/100 g and 1.0 IU/100 g provided the best wound healing rates, with the 0.5 IU/100 g cream inducing 75% healing in 15 days. However, the 1.0 IU/100 g concentration provoked plasma glucose alterations in some animals. In our study, no side effects were observed in the animals, nor were alterations in the glucose levels found 24 h after applying the first dose. Therefore, our dose, minor at 1.0 IU, is safe. However, it is necessary to continue with the research to warrant the security of using insulin as a promotor of wound healing. 

To date, the research regarding the action mechanism of topical insulin in wound healing has involved many aspects, including (i) regulating oxidative responses, decreasing reactive oxygen species that damage lipids, proteins, and DNA [81]; (ii) modulating the inflammatory response, recruiting neutrophils early, and exerting anti-inflammatory effects by increasing M2 macrophages and IL-10 [97]; and (iii) promoting keratinocyte migration, re-epithelialization, and fibroblast activity via the PI3K–Akt–Rac1 pathway, and improving collagen deposition and maturation [98]. Additionally, as we expected, insulin has angiogenic effects, increasing new blood vessel formation, microvascular endothelial cell migration, and endothelial tube formation through PI3K-Akt-SREBP1 signaling and restoring impaired insulin signaling in diabetic wounds by upregulating VEGF and angiopoietin-1 [10,81]. Although the research about insulin as a promotor of wound healing is growing, its use in humans should be considered carefully because of the complexity of determining a dose, administration times, and effects of insulin in possible collateral diseases such as endocrine pathologies and alteration in vessel formation [99].

**Figure 10:**
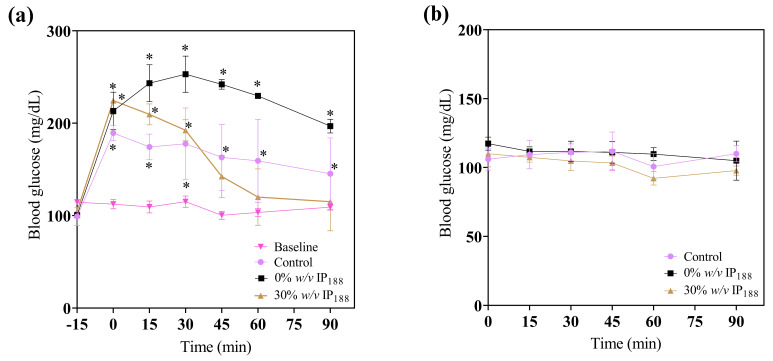
Immediate glucose monitoring in rats after performing the excisional wound. (**a**) Glucose levels 15 min before applying the treatment (−15 min), 0 min at the application of membrane, 15, 30, 45, and 60 min after; (**b**) glucose levels 24 h after the first administration to evaluate the effect of topic insulin in the membrane on glucose in experimental groups. * Difference from basal blood glucose level, *p* ≤ 0.05.

In the treatment application, a significant reduction in glucose (Figure 10) was observed at 45, 60, and 90 min in the 30% *w*/*v* IP_188_ group, which is equal statistically to the baseline group levels. The animals did not present any side effects, such as fainting due to severe hypoglycemia. The blood glucose levels of the 0 and 30% *w*/*v* IP_188_ groups were not statistically different 24 h after the first application from those of the control group. This result could be attributed to the hypoglycemic properties of insulin upon encountering the irrigated margins of the exposed skin, as well as its capacity to stimulate cell proliferation in fibroblasts and keratinocytes, enhance collagen synthesis, promote neovascularization, and ameliorate cellular metabolism through heightened glucose uptake [100,101]. For future therapeutic applications of insulin-containing membranes, it is pertinent to note that the observed reduction in the glucose levels among the groups receiving this treatment may yield beneficial effects for diabetic foot lesions.

The observed hyperglycemia in the experimental groups was attributed to the administration of xylazine/ketamine as an anesthetic agent. Xylazine, an agonist of alpha-2 adrenergic receptors, impacts glucose metabolism by inducing glucagon release, elevating hepatic glucose production, and inhibiting insulin in the pancreas, thereby reducing the tissue insulin uptake [102]. Ketamine, acting as an NMDA (N-methyl-D-aspartate) receptor antagonist, can stimulate the stress response and release catecholamine [103]. Catecholamines, in turn, promote glycogenolysis in the liver, resulting in elevated blood glucose levels [101].

**Figure 11:**
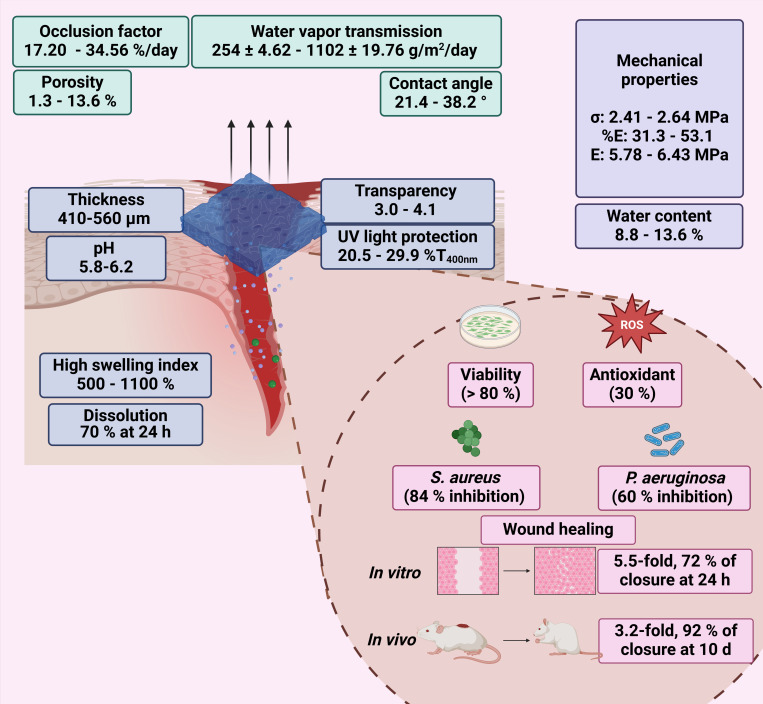
Physical, mechanical, and biological properties of biopolymeric insulin membranes.

This study presented a biopolymeric insulin membrane (0–30% *w*/*v* IP_188_) with suitable physical, mechanical, and biological properties for topical wound dressing application (Figure 11). The membranes exhibited a thickness range of 410–560 µm and adequate physical properties that can be adapted anatomically to wounds. The pH solution was 5.8–6.2, within an acceptable range to avoid skin irritation [48]. The low transmission in the UV range (20.5–29.9% T_400nm_) indicates protection against UV radiation on the wound, while high values in the Vis range (400–800 nm) ensure transparency to monitor the healing process without removing the membranes. Interaction with the wound exudate triggers the swelling of the membrane. Biopolymeric insulin membranes can absorb exudate, presenting a swelling index between 500 and 1100% after 6 h of application. The occlusion capability of the membranes was 17–34% at 24 h, equivalent to regulating the vapor transmission of 2.5 ± 4.6 10^1^–11. 0.2 ± 19.76 × 10^2^ g/m^2^/day. Thus, the membrane could enable the perspiration of wound moisture, indicating the semi-occlusive nature of this wound dressing [42]. A significant dissolution of the membranes was observed after 24 h, with a 70% weight loss, suggesting their potential as resorbable materials. The biopolymeric insulin membranes are low-cost and align with the circular economy concept [28]. Moreover, our biopolymeric insulin membranes demonstrated adequate biocompatibility (>80% cell viability). On the other hand, some factors that compromise wound closure are oxidative stress and wound infections [8]. The 30% *w*/*v* IP_188_ membrane produced a 30% antioxidant activity, similar to the result obtained with the free insulin. Moreover, the membrane caused growth inhibition of *S. aureus* and *P. aeruginosa* by 85% and 75%, respectively. Prominently, the wound healing activity in vitro revealed 72% wound closure at 24 h with an outstanding 3.2-fold increase and 92% in vivo closure at 10 days compared with a placebo with a minimum dose of 0.7 IU in double administration. 

## 4. Conclusions

In this study, we demonstrated the multipurpose potential of insulin delivery in wound repositioning. The insulin was formulated in a biopolymeric membrane of sodium alginate cross-linked with calcium chloride, supported in a mixture of xanthan gum and guar gum, and plasticized with glycerol and sorbitol. The human insulin was combined with poloxamer 188 as a protein stabilizing agent. We demonstrated that our biopolymeric insulin membranes exhibited adequate manipulation and suitable mechanical resistance (2.64 MPa), transparency, pH of topical formulation (5.8–6.2), high swelling capability (1100%), VWTR (1102 g/m^2^/day) with an occlusive factor of 17.20%, and antioxidant activity (30%). Furthermore, the formulation exhibited antibacterial activity (growth inhibition of *S. aureus* at 85% and *P. aeruginosa* at 75%, respectively) and promoted the migration of cells for wound closure (72%) and an increase in the wound closure in vivo of 3.2-fold and 92% closure at 10 days compared with a placebo with a minimum dose of 0.7 IU in a double administration. This is the first study of the insulin formulation in a solid membrane-type pharmaceutical form for wound closure, with integrative and multifunctional properties.

## Figures and Tables

**Table 1:** Physical properties of biopolymeric insulin membranes. Thickness and pH for each IP_188_ membrane.

Membrane	Thickness (µm)	pH
0% *w*/*v* IP_188_	410 ± 0.05	5.84 ± 0.24
1% *w*/*v* IP_188_	450 ± 0.07	6.20 ± 0.08
5% *w*/*v* IP_188_	490 ± 0.05	6.15 ± 0.03
10% *w*/*v* IP_188_	510 ± 0.03	6.24 ± 0.07
30% *w*/*v* IP_188_	560 ± 0.06	5.80 ± 0.07

**Table 2:** Light barrier properties (200 and 400 nm), transparency, and opacity values of membranes.

Membrane	200 nm (% T)	400 nm (% T)	Transparency (% T_600nm_)	Opacity (Abs_500nm_)
0% *w*/*v* IP_188_	7.74 ± 1.98	29.98 ± 4.29	3.92 ± 0.17	0.61 ± 0.04
1% *w*/*v* IP_188_	11.41 ± 0.75	48.41 ± 1.25	3.09 ± 0.10	0.98 ± 0.10 *
5% *w*/*v* IP_188_	18.32 ± 2.73	44.62 ± 1.75	3.53 ± 0.02 *	0.95 ± 0.17
10% *w*/*v* IP_188_	13.71 ± 1.11	40.32 ± 1.02	3.67 ± 0.17	0.76 ± 0.17 ^#^
30% *w*/*v* IP_188_	3.84 ± 0.65	20.53 ± 3.46	4.17 ± 0.02	0.38 ± 0.02

Results are represented as the mean ± SD (*n* = 3). * Statistically significant when compared with 0% *w*/*v* IP_188_ membrane (*p* < 0.05). ^#^ Statistically significant when compared with 30% *w*/*v* IP_188_ membrane (*p* < 0.05).

**Table 3:** Physical and barrier properties of biopolymeric insulin membranes.

Membrane	Water Content (%)	Contact Angle (°)	Porosity (%)	WVTR (g/m^2^/Day)
0% *w*/*v* IP_188_	13.6 ± 1.70	21.48 ± 1.43	13.64 ± 1.35	1102.72 ± 19.76
1% *w*/*v* IP_188_	11.91 ± 1.43	28.82 ± 1.24	3.95 ± 0.51 ^#^	254.64 ± 4.62
5% *w*/*v* IP_188_	10.50 ± 0.60 *	35.29 ± 1.42	2.37 ± 1.45	345.18 ± 4.62
10% *w*/*v* IP_188_	8.84 ± 0.71	38.23 ± 1.71	1.39 ± 0.14	341.95 ± 5.33
30% *w*/*v* IP_188_	11.72 ± 1.03	25.04 ± 1.59	7.50 ± 1.05	1025.75 ± 18.25

Results are represented as the mean ± SD (*n* = 3). * Statistically significant when compared with 0% *w*/*v* IP_188_ membrane (*p* < 0.05). ^#^ Statistically significant when compared with 30% *w*/*v* IP_188_ membrane (*p* < 0.05).

**Table 4:** Inhibition of *S. aureus* and *P. aeruginosa* by biopolymeric insulin membranes.

	*S. aureus*		*P. aeruginosa*	
	24 h	48 h	24 h	48 h
0% *w*/*v* IP_188_^1^	34.7 ± 2.30	0.00 ± 0.70	0.00 ± 2.40	0.00 ± 2.20
1% *w*/*v* IP_188_^1^	0.00 ± 3.70	5.33 ± 5.37	36.59 ± 1.28	74.17 ± 0.33
5% *w*/*v* IP_188_^1^	62.7 ± 1.60	61.10 ± 0.30	69.40 ± 0.90	75.17 ± 0.45
10% *w*/*v* IP_188_^1^	65.00 ± 3.40	62.90 ± 7.80	60.60 ± 2.15	38.99 ± 7.47
30% *w*/*v* IP_188_^1^	84.30 ± 2.40	65.70 ± 2.10	18.28 ± 3.70	12.03 ± 7.03
Poloxamer 188 ^2^	64.11 ± 2.42	41.00 ± 3.40	49.8 ± 1.09	18.45 ± 1.71
Insulin ^3^	33.70 ± 5.3	19.3 ± 1.60	37.82 ± 2.05	57.08 ± 3.25
Clindamycin ^4^	66.62 ± 2.17	66.79 ± 3.91	56.60 ± 0.15	63.28 ± 3.91
Ciprofloxacin ^5^	78.36 ± 0.12	88.09 ± 0.47	97.95 ± 2.04	99.16 ± 0.38

^1^ 15 mg/mL, ^2^ 30% *w*/*v*, ^3^ 50 IU/mL equivalent for membrane concentration, ^4^ 1.5 mg/mg, and ^5^ 50 µg/mL.

## Data Availability

Data are contained within the article and supplementary materials.

## Supplementary Materials

The following supporting information can be downloaded at https://www.mdpi.com/article/10.3390/pharmaceutics16081012/s1, Figure S1: FTIR spectra profile (1000–4000 cm^−1^). (a) Sodium alginate; (b) xanthan gum; (c) guar gum; (d) glycerol; (e) sorbitol; (f) sodium chloride; (g) physical mixture; (h) insulin; and (i) poloxamer 188.; Figure S2: FTIR spectra profile (1000–4000 cm^−1^) of biopolymeric insulin membranes. (a) Vehicle membrane (0 % *w/v* IP_188_), (b) 1 % *w/v* IP_188_ membrane, (c) 5 % *w/v* IP_188_ membrane, (d) 10 % *w/v* IP_188_ membrane, and (e) 30 % *w/v* IP_188_ membrane; Figure S3: Insulin calibration curve; Figure S4: Inhibition of *S. aureus* during the interaction with polymeric insulin membranes. (a) Vehicle membrane (0 % *w/v* IP_188_); (b) 1 % *w/v* IP_188_ membrane; (c) 10 % *w/v* IP_188_ membrane and (d) 30 % *w/v* IP_188_ membrane; (e) ciprofloxacin; (f) clindamycin; (g) sodium alginate; (h) xanthan gum; (i) guar gum; (j) sorbitol; (k) calcium chloride; (l) insulin; (m) poloxamer 188 and; (n) insulin-poloxamer 188 solution. Results are represented as the mean ± SD (*n* = 3). The 5 % *w/v* IP_188_ membrane did not affect growth inhibition; Figure S5: Inhibition of *P. aeruginosa* during the interaction with polymeric insulin membranes. (a) 1 % *w/v* IP_188_ membrane; (b) 5 % *w/v* IP_188_ membrane; (c) 10 % *w/v* IP_188_ membrane and (d) 30 % *w/v* IP_188_ membrane; (e) ciprofloxacin; (f) clindamycin; (g) sodium alginate; (h) xanthan gum; (i) guar gum; (j) glycerol; (k) sorbitol; (l) insulin; (m) poloxamer 188 and; (n) insulin-poloxamer 188. Results are represented as the mean ± SD (*n* = 3). The 0 % *w/v* IP_188_ membrane did not affect growth inhibition; Table S1: Stability of biopolymeric insulin membranes at 4 ° and 27 °C.
