# Supplementary material for: Biopolymeric Insulin Membranes for Antimicrobial, Antioxidant, and Wound Healing Applications
Source: Pharmaceutics. 2024 Jul 30;16(8):1012. doi: 10.3390/pharmaceutics16081012 (PMC11360745; doi:10.3390/pharmaceutics16081012)
Supplement: Supplementary file 1 [file pharmaceutics-16-01012-s001.zip › pharmaceutics-3078788-supplementary.pdf]

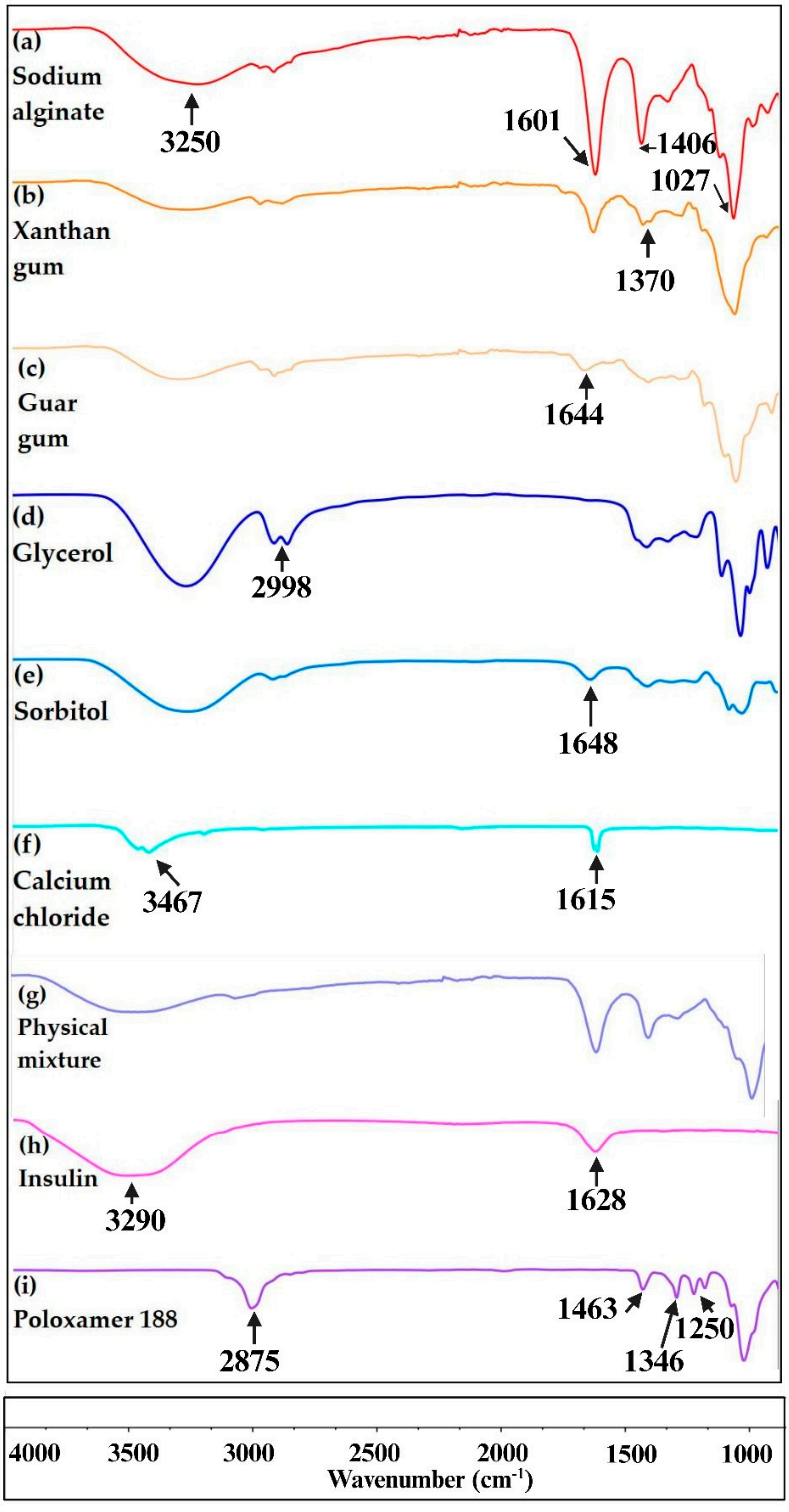


**Figure S1.** FTIR spectra profile (1000 – 4000 $cm^{-1}$). (a) Sodium alginate; (b) xanthan gum; (c) guar gum; (d) glycerol; (e) sorbitol; (f) sodium chloride; (g) physical mixture; (h) insulin; and (i) poloxamer 188.

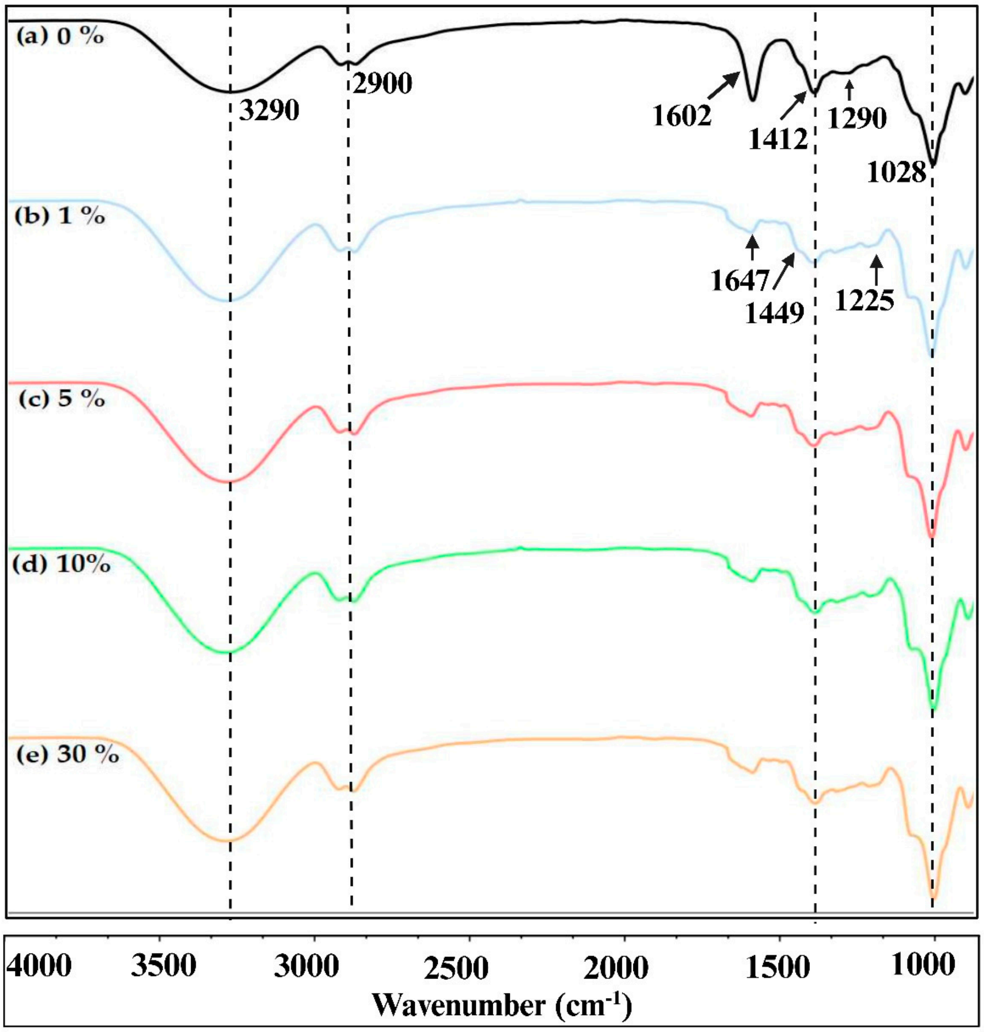


**Figure S2.** FTIR spectra profile (1000 – 4000 $cm^{-1}$) of biopolymeric insulin membranes. (a) Vehicle membrane (0 % *w/v* $IP_{188}$), (b) 1 % *w/v* $IP_{188}$ membrane, (c) 5 % *w/v* $IP_{188}$ membrane, (d) 10 % *w/v* $IP_{188}$ membrane, and (e) 30 % *w/v* $IP_{188}$ membrane.

For insulin quantification, we developed a calibration curve at 0.8 – 3.0 IU/ml interval concentrations at 270 nm (Figure S3). The system's precision was evaluated, obtaining a CV of 0.75 % and deviations of 0.003. For the system linearity test, $r^2 = 0.998$ and IC(β1) =-0.02 ± 0.923 with a detection limit of 0.211 IU/ml and a quantification limit of 0.272 IU/ml. Finally, the reproducibility was obtained with < 1% error.

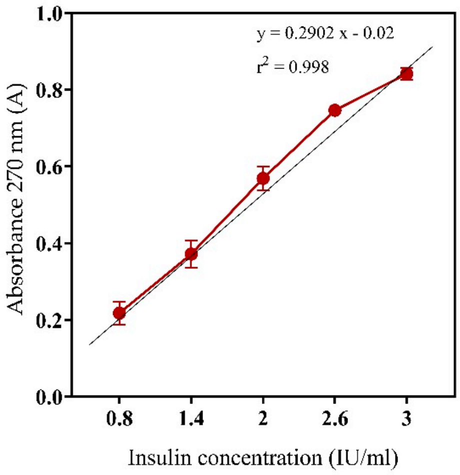


**Figure S3.** Insulin calibration curve.

The 30 % *w/v* $IP_{188}$ membranes were regarded on low (4 ± 2 °C) and high (27 ± 2 °C) temperatures for 24 h, one week, and one month to determine their stability. The membrane was dissolved on 30 ml of PBS (pH 7.4) (dissolution factor), and the absorbance was interpolated on the insulin curve calibration to obtain the insulin concentration (**Table S1**).

**Table S1**. Stability of biopolymeric insulin membranes at 4 ° and 27 °C.

| Time to regard | Insulin concentration (IU/ml) at 4 ± 2 °C | Insulin concentration (IU/ml) at 37 ± 2 °C |
|---|---|---|
| 0 h | 1.67/ 50.1 | 1.40/ 42.0 |
| 24 h | 1.66/ 49.8 | 1.37/ 41.1 |
| 1 week | 1.59/ 47.7 | 1.27/ 38.1 |
| 1 month | 1.58/ 47.4 | 0.88/ 26.4 |

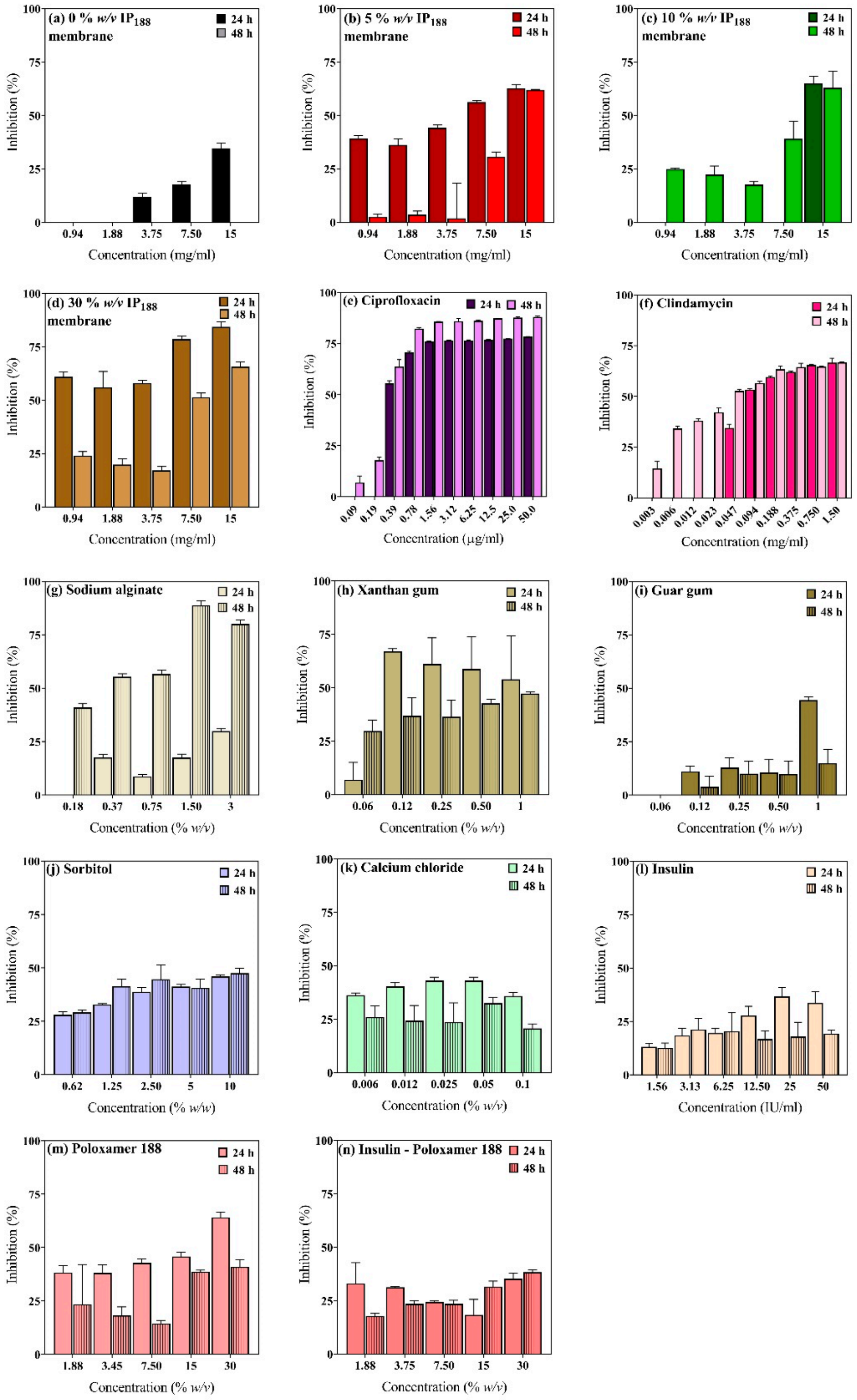


**Figure S4.** Inhibition of *S. aureus* during the interaction with polymeric insulin membranes. (a) Vehicle membrane (0 % *w/v* $IP_{188}$); (b) 1 % *w/v* $IP_{188}$ membrane; (c) 10 % *w/v* $IP_{188}$ membrane and (d) 30 % *w/v* $IP_{188}$ membrane; (e) ciprofloxacin; (f) clindamycin; (g) sodium alginate; (h) xanthan gum; (i) guar gum; (j) sorbitol; (k) calcium chloride; (l) insulin; (m) poloxamer 188 and; (n) insulin-poloxamer 188 solution. Results are represented as the mean ± SD (*n* = 3). The 5 % *w/v* $IP_{188}$ membrane did not affect growth inhibition.

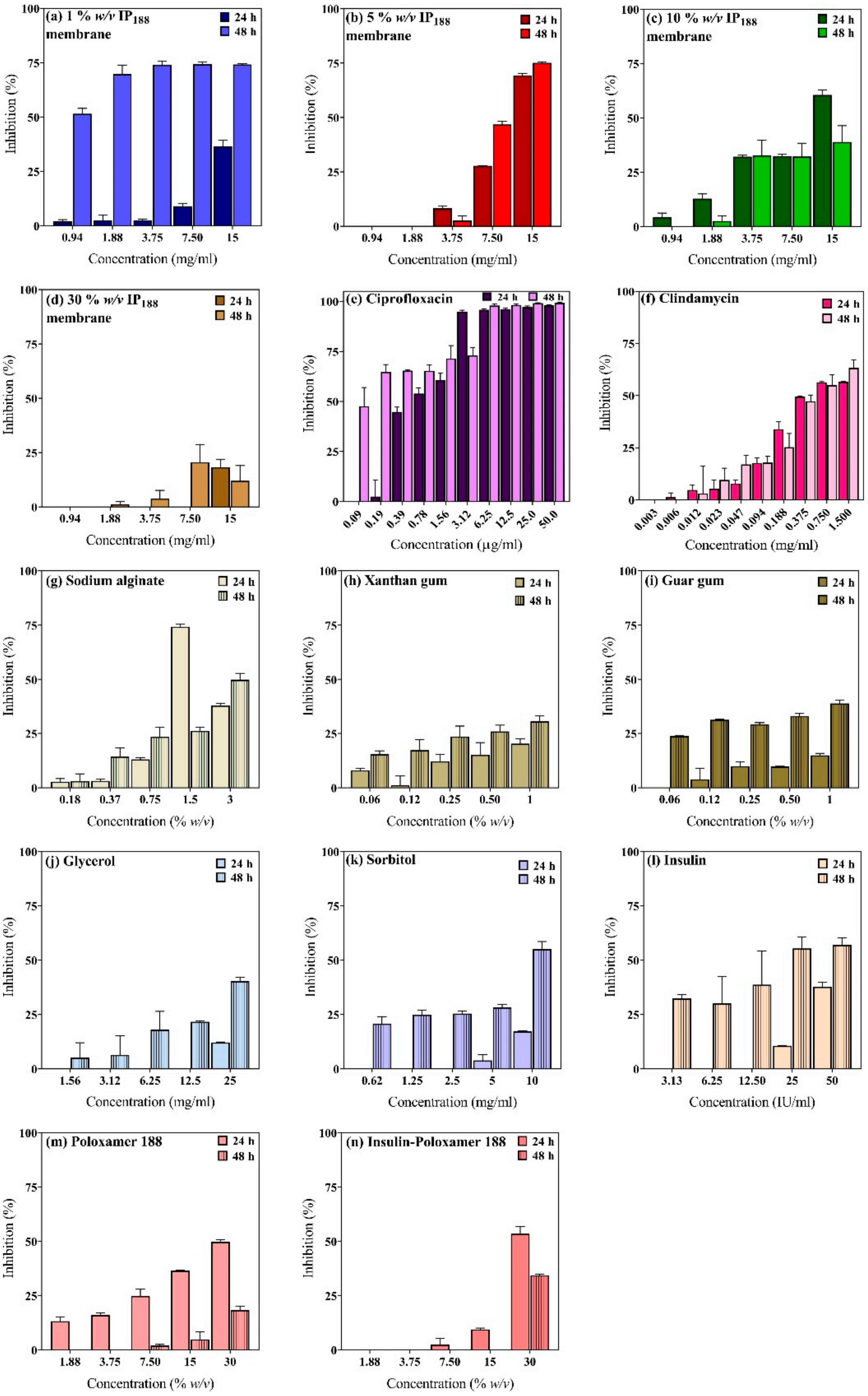


**Figure S5.** Inhibition of *P. aeruginosa* during the interaction with polymeric insulin membranes. (a) 1 % *w/v* $IP_{188}$ membrane; (b) 5 % *w/v* $IP_{188}$ membrane; (c) 10 % *w/v* $IP_{188}$ membrane and (d) 30 % *w/v* $IP_{188}$ membrane; (e) ciprofloxacin; (f) clindamycin; (g) sodium alginate; (h) xanthan gum; (i) guar gum; (j) glycerol; (k) sorbitol; (l) insulin; (m) poloxamer 188 and; (n) insulin-poloxamer 188. Results are represented as the mean ± SD ($n$ = 3). The 0 % *w/v* $IP_{188}$ membrane did not affect growth inhibition.
